# Structural basis of prostate-specific membrane antigen recognition by the A9g RNA aptamer

**DOI:** 10.1093/nar/gkaa494

**Published:** 2020-06-11

**Authors:** Jakub Ptacek, Dong Zhang, Liming Qiu, Sven Kruspe, Lucia Motlova, Petr Kolenko, Zora Novakova, Shambhavi Shubham, Barbora Havlinova, Petra Baranova, Shi-Jie Chen, Xiaoqin Zou, Paloma Giangrande, Cyril Barinka

**Affiliations:** Laboratory of Structural Biology, Institute of Biotechnology of the Czech Academy of Sciences, BIOCEV, Prumyslova 595, Vestec 25250, Czech Republic; Department of Physics and Astronomy, University of Missouri, Columbia, MO, USA; Dalton Cardiovascular Research Center, University of Missouri, Columbia, MO, USA; Department of Internal Medicine, University of Iowa, Iowa City, IA 52242, USA; Laboratory of Structural Biology, Institute of Biotechnology of the Czech Academy of Sciences, BIOCEV, Prumyslova 595, Vestec 25250, Czech Republic; Laboratory of Structural Biology, Institute of Biotechnology of the Czech Academy of Sciences, BIOCEV, Prumyslova 595, Vestec 25250, Czech Republic; Faculty of Nuclear Sciences and Physical Engineering, Czech Technical University in Prague, Brehova 7, Prague 11519, Czech Republic; Laboratory of Structural Biology, Institute of Biotechnology of the Czech Academy of Sciences, BIOCEV, Prumyslova 595, Vestec 25250, Czech Republic; Department of Internal Medicine, University of Iowa, Iowa City, IA 52242, USA; Laboratory of Structural Biology, Institute of Biotechnology of the Czech Academy of Sciences, BIOCEV, Prumyslova 595, Vestec 25250, Czech Republic; Laboratory of Structural Biology, Institute of Biotechnology of the Czech Academy of Sciences, BIOCEV, Prumyslova 595, Vestec 25250, Czech Republic; Department of Physics and Astronomy, University of Missouri, Columbia, MO, USA; Department of Biochemistry, Institute for Data Science and Informatics, University of Missouri, Columbia, MO, USA; Department of Physics and Astronomy, University of Missouri, Columbia, MO, USA; Dalton Cardiovascular Research Center, University of Missouri, Columbia, MO, USA; Department of Biochemistry, Institute for Data Science and Informatics, University of Missouri, Columbia, MO, USA; Department of Internal Medicine, University of Iowa, Iowa City, IA 52242, USA; Laboratory of Structural Biology, Institute of Biotechnology of the Czech Academy of Sciences, BIOCEV, Prumyslova 595, Vestec 25250, Czech Republic

## Abstract

Prostate-specific membrane antigen (PSMA) is a well-characterized tumor marker associated with prostate cancer and neovasculature of most solid tumors. PSMA-specific ligands are thus being developed to deliver imaging or therapeutic agents to cancer cells. Here, we report on a crystal structure of human PSMA in complex with A9g, a 43-bp PSMA-specific RNA aptamer, that was determined to the 2.2 Å resolution limit. The analysis of the PSMA/aptamer interface allows for identification of key interactions critical for nanomolar binding affinity and high selectivity of A9g for human PSMA. Combined with *in silico* modeling, site-directed mutagenesis, inhibition experiments and cell-based assays, the structure also provides an insight into structural changes of the aptamer and PSMA upon complex formation, mechanistic explanation for inhibition of the PSMA enzymatic activity by A9g as well as its ligand-selective competition with small molecules targeting the internal pocket of the enzyme. Additionally, comparison with published protein–RNA aptamer structures pointed toward more general features governing protein-aptamer interactions. Finally, our findings can be exploited for the structure-assisted design of future A9g-based derivatives with improved binding and stability characteristics.

## INTRODUCTION

Prostate-specific membrane antigen (PSMA) is a membrane-tethered glycoprotein naturally expressed in various tissues, including the central nervous system, kidney, small intestine and liver (1–7). At the same time, PSMA is overexpressed on the surface of prostate cancer (PCa) cells compared to the limited expression levels observed in healthy prostate tissue, and this overexpression is significantly correlated to poor disease prognosis (8). The fatality of PCa is mostly caused by progression of the disease to metastatic castration-resistant prostate cancer (mCRPC), with a survival mean of 19 months (9). Moreover, PSMA is overexpressed on the neovasculature of most solid tumors, thus it is an excellent biomarker (10–12). Surprisingly, despite the widespread use of PSMA as a target for PCa imaging and therapy, the role and/or benefit of PSMA overexpression in PCa cells is still poorly understood and the importance of PSMA carboxypeptidase activity for PCa progression is debatable (13–16).

Various approaches targeting PSMA have been developed since 1987 when PSMA was discovered as an antigen specifically recognized by the 7E11-C5 monoclonal antibody (7E11-mAb) raised against LNCaP (a PSMA-positive prostate adenocarcinoma-derived cell line) lysates (17). The ^111^In-radiolabeled 7E11-mAb was later marketed as ProstaScint, the first, and to date the only, PSMA-specific imaging reagent approved by the Food and Drug Administration (17–19). By now, a host of second-generation PSMA-specific antibodies have been prepared and engineered to small antibody fragments, humanized antibodies, radio-labeled derivatives, toxin fusions or bispecific molecules aimed at PCa imaging, diagnosis and therapy (20–24). In addition to antibodies, other modalities targeting PSMA are being developed both as therapeutics and imaging probes, including engineered protein scaffolds, nanoparticles and small-molecule inhibitors (25,26). The latter were originally developed as potential therapeutics for the treatment of neurological disorders by means of inhibiting the enzymatic activity of PSMA residing in the central/peripheral nervous system, also known as glutamate carboxypeptidase II (GCPII) or N-acetylated α-linked acidic dipeptidase (NAALADase) (27,28). These small molecules were later modified with radiotracers or near-infrared fluorophores and are nowadays the most extensively used in the field of prostate cancer imaging and experimental therapy (29,30). Advantages of small-molecule drugs include fast clearance leading to low background, together with the ease of synthesis, but their specificity can pose a challenge as they cross-react with glutamate carboxypeptidase III (GCPIII), a close PSMA homologue (31–33).

Aptamers are synthetic oligonucleotides that utilize a unique three-dimensional fold to target (macro)molecules with high affinity and specificity. Aptamers can be prepared by an *in vitro* selection process, termed SELEX (systematic evolution of ligands by exponential enrichment) (34) and can be used in various biological/biotechnological settings, including the development of biomaterials (35), and as recognition molecules in biosensors and diagnostic, gene-regulatory, therapeutic and imaging applications (36–38). For targeting tumor biomarkers, aptamers offer many beneficial properties. They can be chemically synthesized and modified for imaging or therapeutic purposes (and are not considered biological material), typically bind with high affinities, are nonimmunogenic and feature suitable plasma clearance time for imaging purposes (39,40).

In 2002, Lupold *et al.* used the extracellular part of PSMA to select an aptamer, termed A9, capable of inhibiting PSMA enzymatic activity and binding to PSMA-positive cells with low nanomolar affinity (41). The A9 aptamer, stabilized by the incorporation of 2′-fluor-pyrimidines bases, has since been used in a variety of functional studies, including the A9-mediated delivery of therapeutic siRNA to PCa cells (42), A9-targeted gold nanoparticles for PCa imaging and delivery of doxorubicin payloads (43), and generation of A9-bound drug-loaded liposomes (44). Later, the A9 aptamer was modified to prepare a minimal functional part, giving rise to A9g, its truncated 43-nucleotide variant (45). The A9g variant exhibits a high serum stability (half-life of ∼70 h) and shows no cytotoxicity in human peripheral blood mononuclear cells or undesired immune response in mice, thereby predetermining the A9g aptamer for further *in vivo* studies. Moreover, it seems that its inhibitory activity on PSMA leads to reduced cell migration in culture and suppresses tumors in preclinical mouse models of metastatic PCa (13).

Here, we present a detailed structural, computational and biophysical characterization of interactions between PSMA and A9g. While the performance of the A9g variant in both *in vitro* and *in vivo* assays is fairly satisfactory, we believe that this modality can be advantageously further developed either as a research reagent or to be advanced into the clinic. Data reported here will facilitate the design and characterization of next-generation aptamer variants with improved biophysical and biological characteristics.

## MATERIALS AND METHODS

Unless stated otherwise, all chemicals were purchased from Sigma-Aldrich (St. Louis, MO, USA).

### Cell lines

PC3 and PC3-PIP cells (kindly provided by W. Heston, Cleveland Clinic, Cleveland, OH, USA) (46,47) were grown in RPMI 1640 medium whereas PSMA-positive HEK293T/17 cells (25) and original HEK293T/17 cell line were grown in Dulbecco's modified Eagle medium. All cells were supplemented with 10% v/v FBS and grown under humidified 5% CO_2_ atmosphere at 37°C.

### Site-directed mutagenesis

Plasmids encoding S317A and S317H PSMA mutants were generated by the standard QuikChange site-directed mutagenesis approach using sets of matching mutagenesis primers (Supplementary Table S1) with the pMT/BiP/Strep-FLAG tag/PSMA (for expression of extracellular part of PSMA in insect cells) and pcDNA4/V5-HisA/PSMA (for generation of full length PSMA-overexpressing HEK293T/17 cells) vectors as templates (25). The identity of all clones was verified by Sanger DNA sequencing.

### Generation of stable HEK293T/17 cell lines expressing PSMA

HEK293T/17 cells were seeded in a 12-well plate (TPP, Trasadingen, Switzerland) one day prior to transfection. Expression plasmids encoding individual PSMA variants (wild type PSMA and its S317A and S317H mutants) were transfected into the cells using a jetPRIME transfection reagent (Polyplus-transfection, Illkirch, France) according to manufacturer's protocol at the 1:2 (μg/μl) DNA:jetPRIME ratio. Forty-eight hours post-transfection, cells were transferred to a selection medium containing Zeocin (50 μg/ml, Invivogen, San Diego, CA, USA) and the Zeocin-resistant cell population was selected for additional three weeks. PSMA expression levels of the resulting cell populations were evaluated by flow cytometry using the anti-PSMA 5D3 antibody as described below.

### Heterologous expression and purification of PSMA

The extracellular part of human wild-type recombinant PSMA (wtPSMA; amino acids 44–750) comprising an N-terminal Strep-FLAG tag, as well as its S317A and S317H mutants, were produced as described previously (25). Briefly, the recombinant protein was secreted into the culture medium by stably transfected Schneider's S2 cells. The medium was concentrated by tangential flow filtration (TFF, Millipore, Molsheim, France), dialyzed against the purification buffer (50 mM Tris–HCl, 150 mM NaCl, pH 8.0) and purified by Strep-Tactin affinity chromatography (Strep-Tactin Superflow, IBA, Germany) followed by size exclusion chromatography on a Superdex HR200 column (GE Healthcare, Chicago, IL, USA) in TBS buffer (50 mM Tris–HCl, 150 mM NaCl, pH 7.4).

N-terminally-tagged (Avi-tag biotinylated *in-vivo*) murine PSMA (mPSMA) and human GCPIII were recombinantly expressed from Drosophila S2 cells and affinity purified using the Streptavidin Mutein Matrix (Roche, Basel, Switzerland). Detailed protocols for cloning, expression and affinity purification of the PSMA paralogs/orthologs are described elsewhere (48,49). The final size-exclusion chromatography step was identical as described above for human PSMA.

### Aptamer synthesis and refolding

A9g and Alexa Fluor647-A9g aptamers were commercially synthesized by TriLink Biotechnologies (San Diego, CA, USA). Both aptamers were stabilized by the incorporation of 2′-deoxy-2′-fluororibose-pyrimidine bases replacing the natural nucleotides. Aptamer purity and identity was determined by polyacrylamide gel electrophoresis and mass spectrometry analysis, respectively. The aptamers were refolded by the dilution into a refolding buffer (10 mM aptamer; 20 mM HEPES, 150 mM NaCl, 2 mM CaCl_2_), the solution was heated to 95°C and allowed to cool to room temperature at a rate of 1°C/min.

### Crystallization

Initial sparse-matrix crystallization screening was performed against several commercially available crystal screens using a Gryphon robot (Art Robbins, Sunnyvale, CA) in a sitting-drop vapor-diffusion setup at room temperature. The most promising conditions were further refined, and the diffraction quality crystals were grown in hanging drops formed by mixing equal volumes of PSMA, A9g, and 2-(phosphonomethyl)-pentanedioic acid (2-PMPA) at the 1:1.1:14 molar ratio (final concentrations: 5.8 mg/ml PSMA, 80 μM A9g, 1 mM 2-PMPA) and the optimized crystallization solution (30% [v/v] MPD, 0.2 M magnesium acetate, pH 4.6) at room temperature. Rod-shaped crystals (the *P*4_1_2_1_2 space group) of approximate dimensions of 120 × 40 μm grew within one week. For data collection, crystals were vitrified in liquid nitrogen directly from crystallization droplets.

### Data collection, structure solving, refinement and analysis

Diffraction data were collected at Helmholtz-Zentrum Berlin (BESSY-II) at the 14.1 beamline at 90 K. The diffraction images were processed using DIALS mode (50) in Xia2 (51). The phase problem was solved by molecular replacement using the PHASER Program (52) and the structure of free PSMA (PDB code 2OOT) (53) as a search model. The model of the aptamer was built using the RCRANE (54) tool in COOT (55). The structure was refined using REFMAC5 with 5% of reflections used as the testing (free) set. The last cycle of the refinement was performed using all reflections. Overall, quality of the structure was evaluated using the MOLPROBITY validation tool (56). A Ramachandran outlier was observed for the residue V382. Inspection of several crystal structures of PSMA solved at high resolution (<1.5 Å; PDB codes 5OF0 and 5O5T (57)) also revealed the presence of this outlier, thus corroborating the correct modeling of this residue in the PSMA/A9g complex. The atomic coordinates and structure factors have been deposited in the PDB under the code 6RTI. The data collection and refinements statistics are shown in Supplementary Table S2.

### IC_50_ determination

Inhibition of PSMA enzymatic activity by the A9g aptamer was determined using a radioenzymatic assay with [^3^H]NAAG (radiolabeled at the terminal glutamate) as described previously (58). Briefly, PSMA (20 ng/ml) was preincubated in the presence of increasing concentrations of the aptamer in 20 mM Tris, 150 mM NaCl, pH 8.0, for 10 min at 37°C in a total volume of 80 μl. The reaction was initiated by addition of 40 μl of a mixture of 0.31 μM NAAG and 15 nM [^3^H]NAAG (50 μCi/mmol in Tris buffer, Perkin Elmer) to the total reaction volume of 120 μl. After 20 min, the reaction was terminated with 120 μl of 200 mM potassium phosphate, 50 mM EDTA, 2 mM β-mercaptoethanol, pH 7.4. The released glutamate was separated from the reaction mixture by ion-exchange chromatography and quantified by liquid scintillation. Duplicate reactions were carried out for each experimental point. Data were fitted using GraphPad Prism software (GraphPad Software, San Diego, CA, USA), and IC_50_ values were calculated from the inhibition curves.

### Flow cytometry

Tris-buffered saline (TBS; 10 mM Tris, 150 mM NaCl, pH 7.4) supplemented with 0.5% (w/v) gelatin from cold water fish skin was used for all washes and incubations. Cells were detached by solution of trypsin (0.025% w/v) and EDTA (0.01% w/v) in PBS for 3 min, washed and preincubated with 1 μM inhibitors (RNA 2–65, EPE and 2-PMPA in case of a competition experiment) for 15 min at 4°C (inhibitors were omitted from experiments analyzing A9g interactions with S317A and S317H mutants). The A9g aptamer conjugated to Alexa Fluor647 (100 nM final concentration) was added to the cell suspension and incubated for 15 min. Cells were washed and treated with Hoechst 33258 to calculate viability. At least 50 000 cells per sample were immediately analyzed by the LSRFortessa flow cytometer (BD Biosciences, San Jose, CA, USA). Data were processed in FlowJo software (FlowJo, LLC, Ashland, OR, USA).

To analyze surface expression levels of PSMA mutants, the 5D3 monoclonal antibody (21) was used at 40 nM concentration and incubated with cells on ice for 20 min. After washing with PBS supplemented with 0.5% (w/v) gelatin, cells were labeled with goat anti-mouse secondary antibody conjugated with Alexa Fluor647 (Invitrogen, Carlsbad, CA; 4 μg/ml) on ice for 20 min. Further washing, labeling, flow cytometry and analysis steps were performed as described above.

### Differential scanning fluorimetry (DSF)

The extracellular part of PSMA (wild-type and S317A and S317H variants) was diluted to 0.1 mg/ml in TBS. 2-PMPA and/or A9g were added in 10-fold molar excess and incubated for 10 min at 4°C (in case of samples with both 2-PMPA and A9g, the 2-PMPA was added first and preincubated for 5 min). Thermostability was measured on Prometheus NT.48 (NanoTemper Technologies GmbH, München, Germany) using standard capillaries. Following a 5 min stabilization step at 20°C, the temperature was gradually increased by 1°C/min up to 95°C and fluorescence at λ_ex_ = 280 nm and λ_em_ = 330 and 350 nm was detected. The melting curve was plotted as a ratio of fluorescence at 350/330 nm versus temperature and the melting temperature was determined at the inflection point of the melting curve.

### Computational modeling of the free state of the A9g aptamer

**Figure 1.**
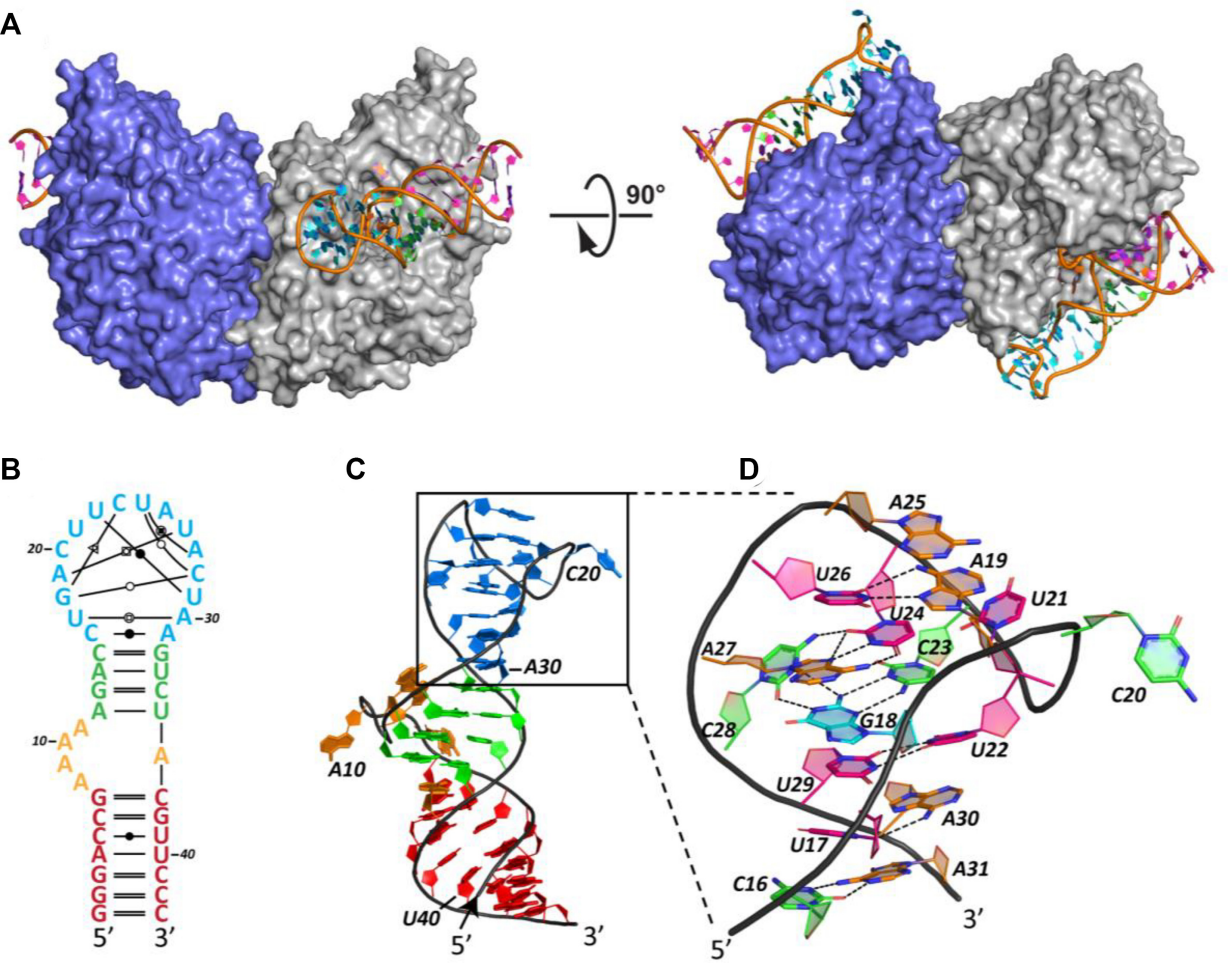
Experimentally determined structure of PSMA-bound A9g aptamer. (**A**) Overall structure of the PSMA/A9g complex. PSMA homodimer is shown in surface representation with individual monomers colored blue and gray, respectively. A9g binds PSMA with the 1:1 stoichiometry. (**B**) Schematic representation of A9g adopting a simple step-loop fold that can be divided into four parts – S1 stem (7 nucleotides; red), 4 × 1 L1 loop (adenines A8-A11 on the 5′ strand and A36 on the 3′ strand; orange), S2 stem (4 nucleotides; green) and the L2 hairpin loop (C16 through A31; blue). The noncanonical base pairing in the L2 loop was assigned by RNApdbee (86) and denoted 
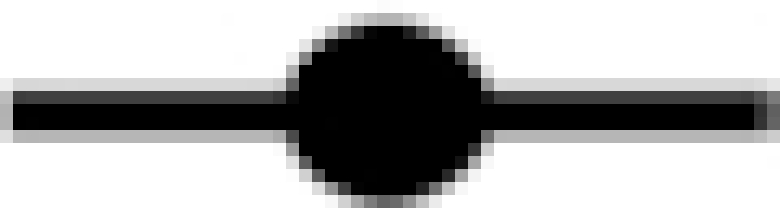
cis Watson–Crick Watson–Crick, 
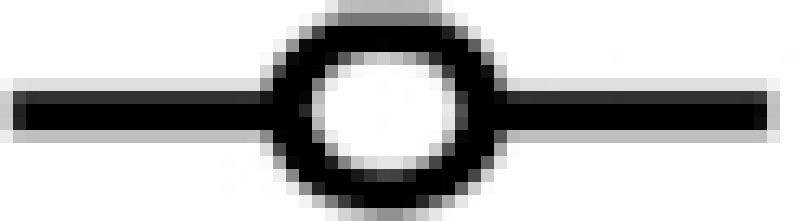
trans Watson–Crick Watson–Crick, 
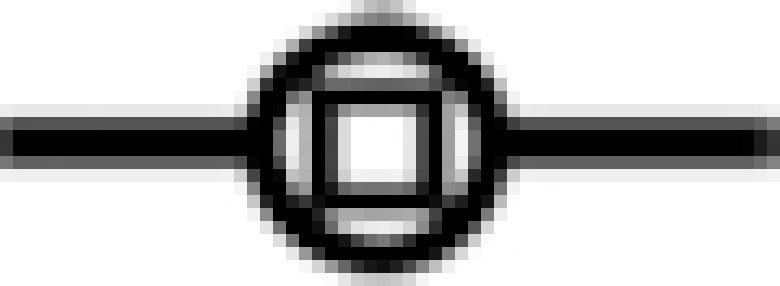
trans Watson–Crick Hoogsteen, 
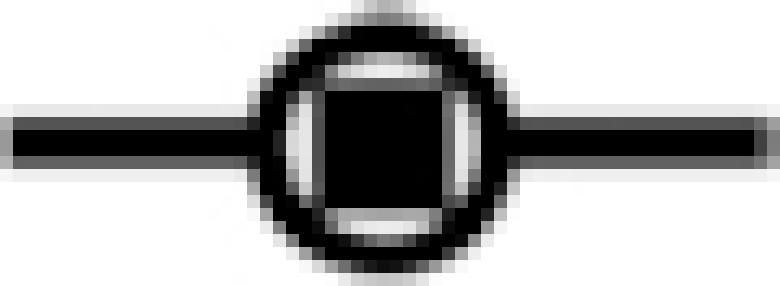
cis Watson–Crick Hoogsteen, 
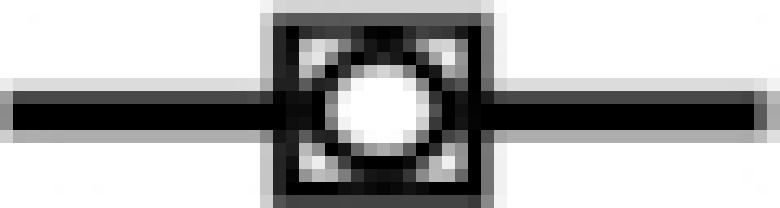
trans Hoogsteen Watson–Crick, 
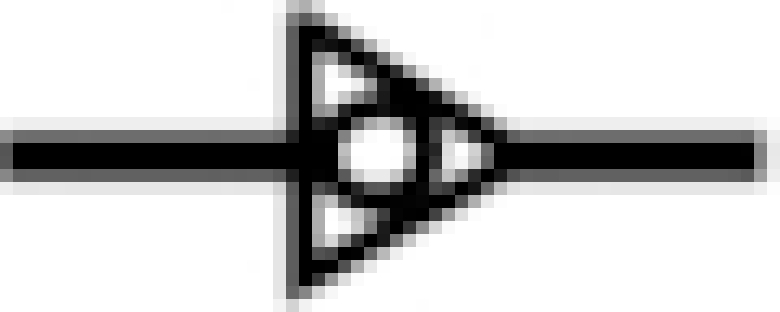
trans Sugar Watson–Crick. (**C**) 3D structure of A9g (color-coding as in panel B). The sharp turn of RNA backbone of the L1 loop causes flipping of the four adenines out from the helical core. (**D**) Detailed view of the 3D arrangement of the L2 loop. Network of eight noncanonical base pairs plays a crucial role in stabilization of the hairpin loop conformation upon PSMA binding.

Using the PSMA-bound A9g aptamer structure as the initial state, we performed replica-exchange molecular dynamics (REMD) simulations with the IsRNA coarse-grained model (59) to predict the free A9g aptamer solution structure. Specifically, we ran Langevin dynamics simulation with temperatures uniformly ranging from *T* = 200 to 425 K (10 replicas total). With the experimental structure of A9g in the complex with PSMA in hand, we noted that the computationally predicted secondary structure (60) is nearly identical to that of the bound A9g. Therefore, we used the Watson–Crick base pairs shown in Figure 1B as constraints in the REMD simulations. The simulation time for each replica was *t* = 50 ns with an integration time step Δ*t* = 1 fs. After the first 25 ns of simulation that allowed for the relaxation from the bound A9g structure, we collected structure snapshots every 50 ps for the remaining 25 ns simulation trajectories for all the replicas.

From the simulations, a conformational ensemble of 5100 structures was generated for the free state. To obtain the final predicted structures from the conformational ensemble, a conventional ‘energy cutoff and structure clustering’ strategy was used (61,62). In our calculation, the top 10% lowest-energy structures in the ensemble were selected and clustered. In the clustering procedure, we defined any two structures with pairwise root-mean-square deviation (RMSD) less than a threshold value of 5.0 Å as a neighbor. We then identified the structure with the largest number of neighbors as the first centroid structure. The first centroid structure and its neighbors were clustered as the first (largest, i.e., most populated) cluster and the first centroid structure is predicted as the top 3D structure. Applying the same approach to the remaining conformations resulted in additional (smaller, i.e. less populated) clusters and the corresponding centroid structures. Our clustering procedure resulted in 10 ranked main clusters and 10 corresponding predicted 3D centroid structures. Specifically, the first three clusters contain 69, 61, and 38 conformations, respectively.

## RESULTS

### PSMA/A9g complex—crystallization and structure solution

The extracellular part of human PSMA (amino acids 44–750) was co-crystallized with A9g and 2-(phosphonomethyl)-pentanedioic acid (2-PMPA) at the 1:1.1:14 molar ratio. 2-PMPA is a reversible PSMA-specific inhibitor competing with the substrate in the active site of PSMA with the inhibition constant in a picomolar range. 2-PMPA was included in the crystallization droplets as our previous experiments revealed that the presence of the inhibitor greatly increases the probability of obtaining diffraction-quality crystals (63). The structure was phased by molecular replacement using PSMA coordinates of unliganded PSMA (PDB code 2OOT) (53) as the search model. The final model was refined to the 2.2 Å resolution limits with the crystallographic factors *R* = 19.2% and *R*_free_ = 21.7%, and good stereochemical parameters (Supplementary Table S2). High resolution allowed us to trace the entire A9g aptamer sequence. The final electron density map for the A9g aptamer complex is of excellent quality (the composite omit map with simulated annealing (64) is shown in Supplementary Figure S1). The crystallographic asymmetric unit contains a single PSMA/A9g/2-PMPA complex and the physiological PSMA dimer was created using the two-fold symmetry operator (Figure 1A).

### Structural features of the PSMA-bound A9g aptamer

The experimentally determined fold of the 43-nucleotide A9g aptamer in its PSMA-bound state is a relatively simple stem-loop structure that includes two stems termed S1 (7-base pairs) and S2 (4-base pairs), a 4 × 1 internal loop 1 (L1; 4 unpaired adenines A8–A11 on the 5′-side and an unpaired nucleotide A36 on the 3′-side) and a 16-nucleotide long hairpin loop 2 (L2; nucleotides C16–A31; Figure 1B and C).

The two S1 and S2 helix stems are coaxially stacked via nucleotide A36 (Figure 1C). All four unpaired adenine nucleobases (A8–A11) in the L1 loop are flipped out of the double helical central stem and engage residues of PSMA, and the corresponding backbone makes a sharp turn that allows its insertion into the PSMA internal cavity. Interestingly, a network of eight noncanonical base pairs is observed in the L2 loop, and residues U22 through U24 of the L2 loop fold back to interact with residues G18, A27, C28 and U29. The structure of L2 is further stabilized by an extensive network of additional hydrogen bonding interactions (Figure 1D and Supplementary Figure S2).

### Computational modeling of the free A9g aptamer

**Figure 2.**
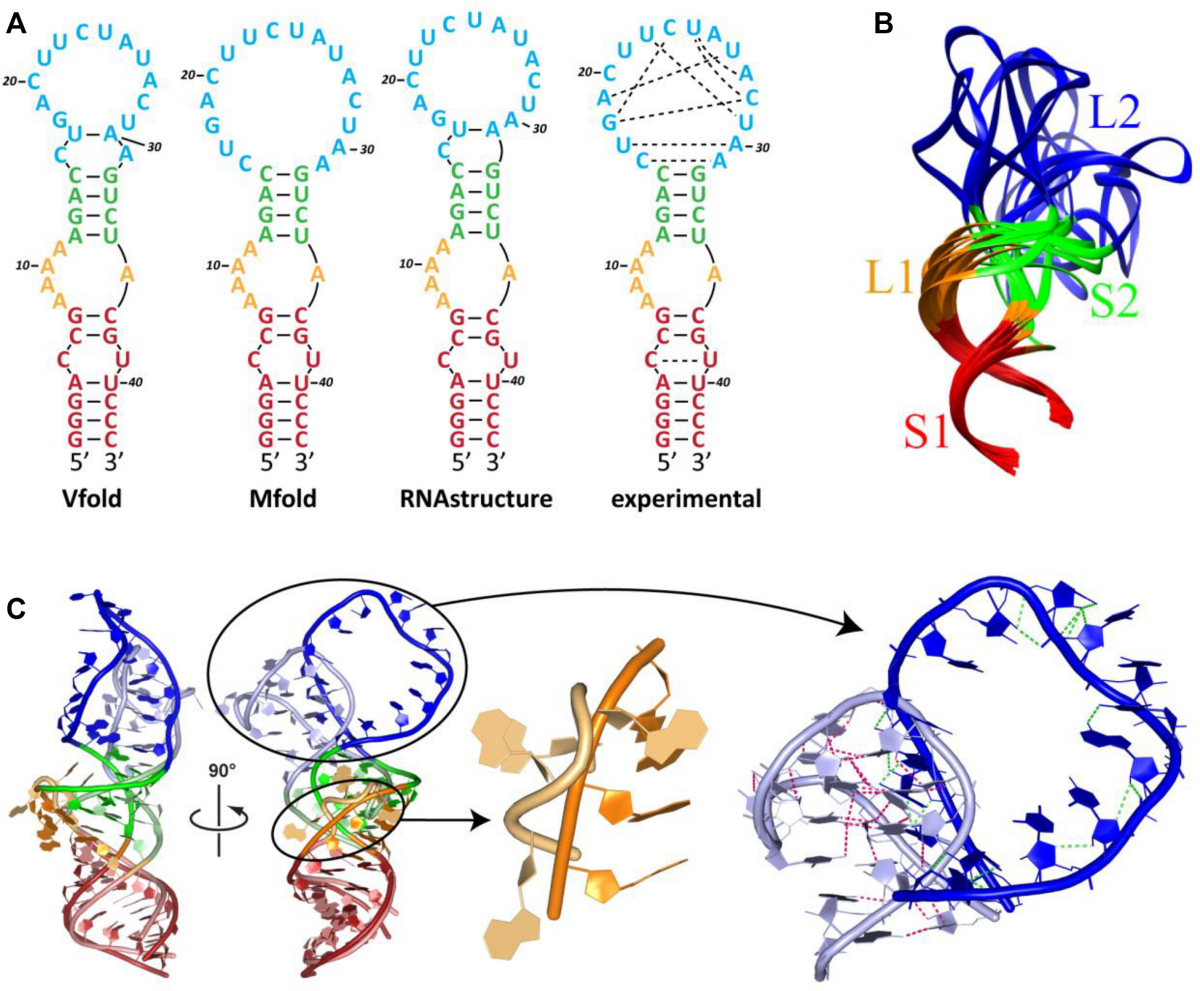
Comparison of *in silico* models of free A9g with the experimentally determined structure of the PSMA-bound aptamer. (**A**) Secondary structure predictions of the free A9g aptamer by the free energy-based programs Vfold, Mfold and RNAstructure, compared to the experimentally determined structure reported here. Predicted structures are colored red, orange, green and blue for S1 stem, L1 loop, S2 stem and L2 loop, respectively. Canonical and noncanonical base pairing is shown in full and dashed lines, respectively. (**B**) The top 10 predicted centroid structures for the A9g free state. The noncanonical base pairing in the PSMA-interacting L2 loop is disrupted in solution and the loop is quite flexible. (**C**) 3D structures of the bound (experimental) and free (*in silico* modeled) A9g. Superposition of the top predicted structure of the free A9g aptamer (dark) and the experimental 3D structure (light). While the overall spatial organization is conserved, there are noticeable structural differences in L1 and L2 loops interacting with PSMA. In the absence of intermolecular RNA–protein interactions, the four unpaired adenines A8–A11 of the L1 loop preferably flip in to form energetically more favorable stacking interactions. A noticeable rearrangement of intramolecular polar interactions within the L2 loop occurs upon A9g binding to PSMA as compared to the free aptamer (magenta and green dashed lines, respectively).

To understand conformational changes of A9g upon PSMA-binding, we used REMD simulations in the coarse-grained IsRNA model (59) to model the A9g solution structure. *In silico* models of free A9g, as an output of the free energy-based programs Vfold (60), Mfold (65) and RNAstructure (66), consistently predicted a stable A9g fold with the pattern of the Watson–Crick base pairs being virtually identical to that extracted from the experimental PSMA-bound structure (Figure 2A). With the Watson–Crick base pairs of the experimentally determined aptamer structure used as constraints, the simulations further revealed that the noncanonical pairing in the L2 loop of the experimental PSMA-bound state is easily disrupted in solution and the L2 loop can adopt various conformations to maximize its entropy (Figure 2B). At the same time, upon PSMA binding, only a single L2 conformation of the large free-state ensemble is stabilized. These findings suggest that (a) the specific noncanonical interactions in the (bound-state) loop require the presence of the PSMA and (b) the intra-loop interactions in the aptamer and the PSMA–aptamer interactions act in cooperation. Thermodynamically, in the free aptamer, the absence of PSMA interactions is compensated for by the enhanced conformational entropy (flexibility). However, upon PSMA binding, a single PSMA/aptamer conformation is selected and stabilized.

Similarly, in contrast to the flipped-out conformation of the four unpaired adenines A8–A11 of the L1 loop in the PSMA-bound structure, these nucleobases are flipped in in the free state and are engaged in the successive stacking, which is more energetically favorable. Since the nucleotides A8-A11 serve as the PSMA-binding motif, A9g binding to PSMA likely elicits changes in the dynamics of the L1 loop, leading to the disruption of the stacking between the four adenine nucleobases and the subsequent energetically unfavorable distortion of the backbone.

Overall, the computational modeling implies that A9g retains its stem–loop–stem–loop secondary structure both in solution and in the complex with PSMA. Compared to stable conformations of stems, the loops are more prone to structural changes upon PSMA binding, as witnessed by the disruption of stacking interactions and base flipping in the L1 loop and a rearrangement of base pairing observed in the L2 loop (Figure 2C).

### Overview of the PSMA/A9g interaction interface

Two A9g molecules bind symmetrically to the PSMA homodimer, i.e. the binding epitope and interface are identical for each PSMA monomer, and reveal an extensive interaction with PSMA, which buries 3096 Å^2^ of the combined (A9g + PSMA) accessible surface area for each aptamer/PSMA interface (calculated using the PISA software (67); Figure 1A). On the aptamer side, 25 of 43 residues interact with PSMA and the interaction interface accounts for 19% of the total solvent accessible area of A9g. With respect to PSMA, 47 residues (out of 692) engage A9g, covering 6% of the total solvent accessible area.

Within the crystal lattice, crystallographic contacts of the PSMA/A9g/2-PMPA complex are, among others, mediated via stacking interactions of bases of G1 of the symmetry mates. Additionally, the crystal packing together with the symmetry operators could produce an alternative binding PSMA/A9g interface, although with a substantially smaller buried surface area of 1218 Å^2^ (Supplementary Figure S3). However, as the alternative A9g binding mode is inconsistent with our mutagenesis, kinetic and cell-based experiments (see below), we are confident that the presented PSMA/A9g interface is the one existing in solution.

### Details of the PSMA/A9g interaction interface

**Figure 3.**
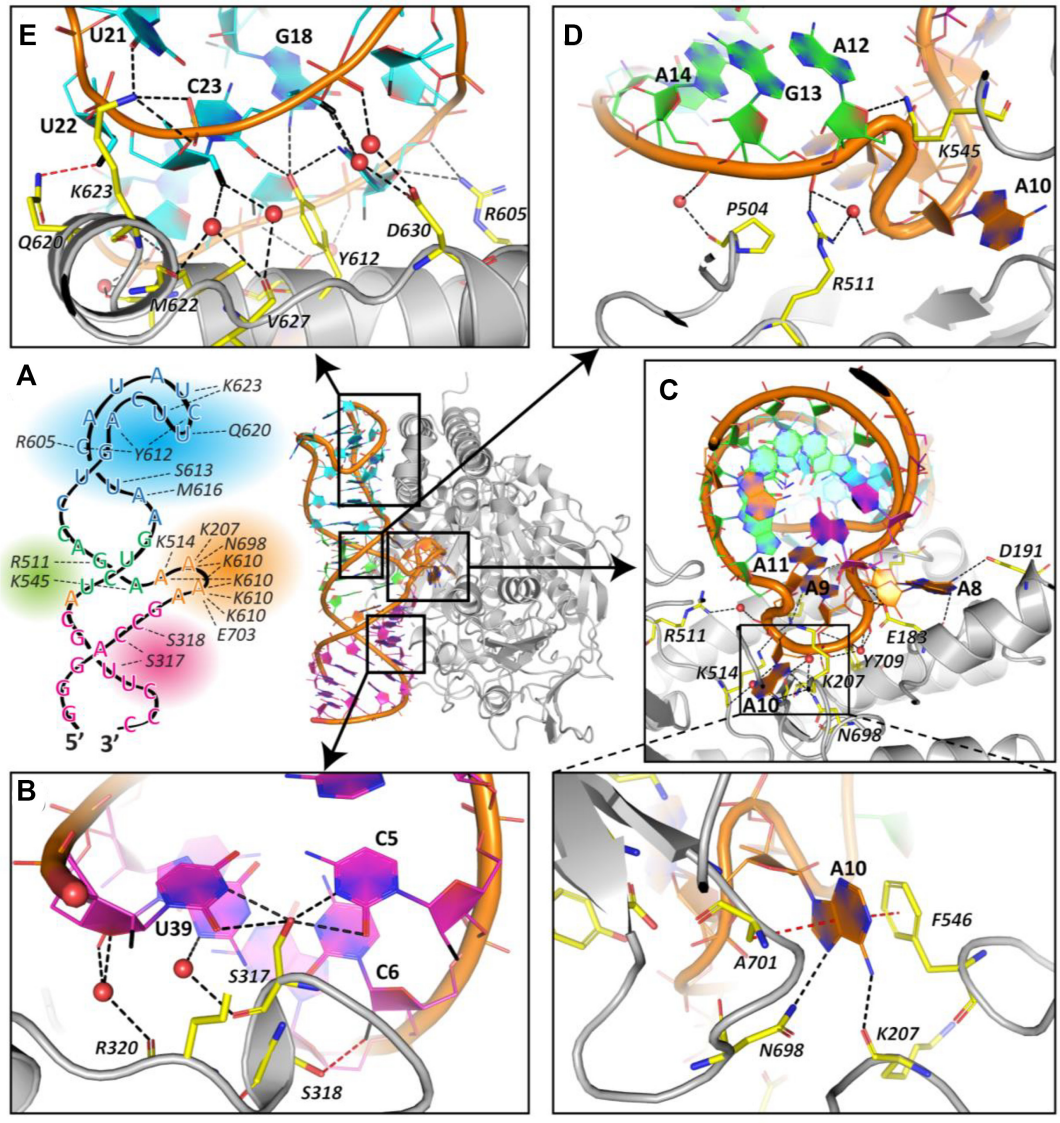
Structural characterization of the PSMA/A9g complex. The A9g backbone is shown as an orange ribbon, nucleotides are represented by lines with filled-in rings and are color-coded based on the secondary structure assignment – S1 stem (magenta), L1 loop (orange), S2 stem (green) and L2 loop (cyan). Hydrogen bonds are shown as dashed lines, water molecules as red spheres. (**A**) A schematic 3D representation of A9g together with amino acids involved in direct hydrogen-bonding interactions with PSMA (left) and a structural ‘preview’ of the PSMA/A9g complex (right). (**B**) Detailed view of the S1 stem interaction interface. S317 (yellow stick) is the key PSMA-interacting residue bridging nucleobases of C5 and U39. Also shown is a noncanonical hydrogen bond between the C6 fluorine atom (black) and the S318 hydroxyl group (red dashed line). (**C**) The L1 loop is flipped out of the double helical secondary structure of the S1 and S2 stems. The A10 nucleobase is inserted into the entrance funnel of PSMA, where it is stacked between side chains of F546 and A701 and furthermore is hydrogen-bonded to the carbonyl oxygen of K207 and the side chain amide of N698. It should be noted that N698 and A701 are part of the glutarate sensor, a flexible amino acid segment critical for substrate recognition. (**D**) Limited S2-PSMA interface is formed by two direct and two water-mediated H-bonds. (**E**) The L2 hairpin loop interacts extensively with residues of the C-terminal domain of PSMA. A wide network of hydrogen bonds is shown by dashed lines and residues Y612, K623 and R605 contribute with 8 of 10 total direct hydrogen bonds. A noncanonical hydrogen bond between the U22 fluorine atom (black) and the Q620 side chain amide is shown as a red dashed line.

The schematic 3D representation of A9g together with amino acids involved in direct hydrogen-bonding interactions with PSMA residues and the corresponding structural ‘preview’ of the PSMA/A9g complex are shown in Figure 3A. Both the schematic representation and the experimental structure of A9g are color-coded based on its four structural motifs that are spatially separated and correlate with the aptamer secondary structure: the S1 stem (magenta), L1 loop (orange), S2 stem (green) and L2 loop (blue).

At the S1 stem-PSMA interface, a short S317–R320 amino acid stretch engages the major groove of S1. Here, the S317 is of critical importance as its hydroxyl group bridges U39 and C5 nucleobases, the only non-Watson–Crick base pair in S1, with interatomic distances of 2.8 Å (N3) and 3.2 Å (O2) of U39, and 3.0 Å (O2) and 2.6 Å (N3) of C5 (Figure 3B and Supplementary Figure S4A). Previous studies have implicated U39 as a critical component of the PSMA/A9g interface, and the mutation of U39 to G39 markedly decreased A9g affinity for PSMA (45). Our findings provide a mechanistic rationale for these observations. While the U39G substitution would likely stabilize the overall 3D fold of the free aptamer, this substitution would at the same time form a steric barrier, hindering PSMA/A9g binding (Supplementary Figure S4B). In addition to the direct canonical hydrogen bonds, two water-mediated H-bonds between the carbonyl oxygen of Ser317 and N3 of G38 and carbonyl oxygen of R320 and O4 of U39 and O2 of G38 contribute to the S1-PSMA interaction interface. Ribose moieties of all cytidine and uridine nucleotides of A9g are replaced by the 2′-deoxy-2′-fluororibose to enhance the stability of A9g against RNase degradation. Given its high electronegativity, the fluorine atom can, in principle, function as a hydrogen bond acceptor (68,69). One of the two such examples in the PSMA/A9g complex is a noncanonical hydrogen bonding between the carbonyl oxygen of S318 and the fluorine atom of the C6 ribose (3.0 Å; Figure 3B).

The seven nucleotide-long S1 stem is terminated by the L1 loop (A8 − A11 + A36), where the A9g backbone makes a tight turn that projects the A10 nucleobase deep into the internal substrate-binding pocket of PSMA (Supplementary Figure S5). Here, the A10 adenine ring is stacked between the phenyl ring of F546 (3.6 Å) and the side chain of A701 (3.8 Å) and is further hydrogen-bonded to the main chain carbonyl of K207 (3.0 Å) and the ND2 nitrogen of N698 (3.2 Å; Figure 3C). It is interesting to note that F546 and A701 are located at the entrance lid and the glutarate sensor, respectively, the two highly flexible segments of PSMA that are critical for substrate hydrolysis by PSMA (70,71). A8 and A9 (both nucleobase rings and the RNA backbone) also form an extensive hydrogen-bonding network, both direct and water-mediated, with several PSMA residues, including K514, K610, E703 and Y709 (Figure 3C). Moreover, nucleobase rings of A8 and A9 engage PSMA nonpolar surface patches formed around side chains of F186 and I614, respectively (Supplementary Figure S6).

Interactions between the short S2 stem (four base pairs) and PSMA are quite limited and comprise only two direct and two water-mediated H-bonds of the A9g backbone and residues P504, R511 and K545 (Figure 3D). In contrast, the L2 loop, similarly to the L1 loop, has the most extensive interface with PSMA involving nucleobases and the backbone of 10 nucleotides. Unlike the L1 loop protruding into the internal binding pocket of PSMA, the L2–PSMA interface is fairly flat and encompasses a rectangular arrangement of three α-helices (α15-loop-α16-loop-α17) of the C-terminal domain of PSMA (70). Here, 10 amino acids form a network of direct and water-mediated hydrogen bonds with A9g, with the noticeable involvement of the side chains of Y612, K623 and R605 that contribute with eight out of 10 direct H-bonds. It is worth noting that one direct and two water-mediated noncanonical hydrogen bonds are observed for fluorine atoms of U22 (Q620 Nϵ2; 3.0 Å) and C23, respectively (Figure 3E).

The PSMA–A9g complex was crystallized using a precipitant solution of pH 4.2. Consequently, it is likely that isolated local features at the PSMA–A9g interface can differ from interactions existing at the physiological pH due to a different protonation state of carboxylic functional groups of glutamate and aspartate side chains. For example, while a direct hydrogen bond was modeled between the protonated side chain of E183 and the backbone phosphate of A8, upon conformational rearrangement this interaction would be likely mediated by a metal ion (e.g. Na^+^, K^+^ or Ca^2+^) or a water molecule under physiological conditions.

Collectively, the extensive A9g–PSMA interaction interface is predominantly formed by L1 and L2 loops of A9g, although of critical importance is also S317 of PSMA, which is inserted between nucleotides C5 and U39 of the S1 stem. There is an interesting distinction between binding modes of the two loops: while the L1 loop, adopting a tight U-turn, is inserted into the entrance funnel leading to the active site of PSMA, the L2 loop follows a contour of the PSMA flat surface.

### PSMA structural changes upon A9g binding

Despite the extensive set of PSMA/A9g interactions, inspection of the PSMA/A9g complex shows no fundamental changes in the overall fold of the enzyme and observed conformational changes are mostly localized at the PSMA/A9g interface. This fact is best documented when our ternary complex is compared to the PSMA/2-PMPA complex (PDB code: 2PVW) published previously (70), where the catalytic core and virtually all secondary structure elements occupy nearly identical positions in both structures. Limited changes are observed only for several surface loops and also for the α16 helix (amino acids 618–625) interfacing with the L2 loop, which is repositioned by ∼1 Å. Quantitatively, Cα- and heavy-atom RMSDs are 0.44 Å and 0.78 Å, respectively, for the whole protein (684/5439 corresponding Cα/heavy atoms), compared to Cα- and heavy-atom RMSDs of 0.94 Å and 1.45 Å, respectively, for residues at the A9g-interacting interface (42/356 corresponding Cα/heavy atoms).

**Figure 4.**
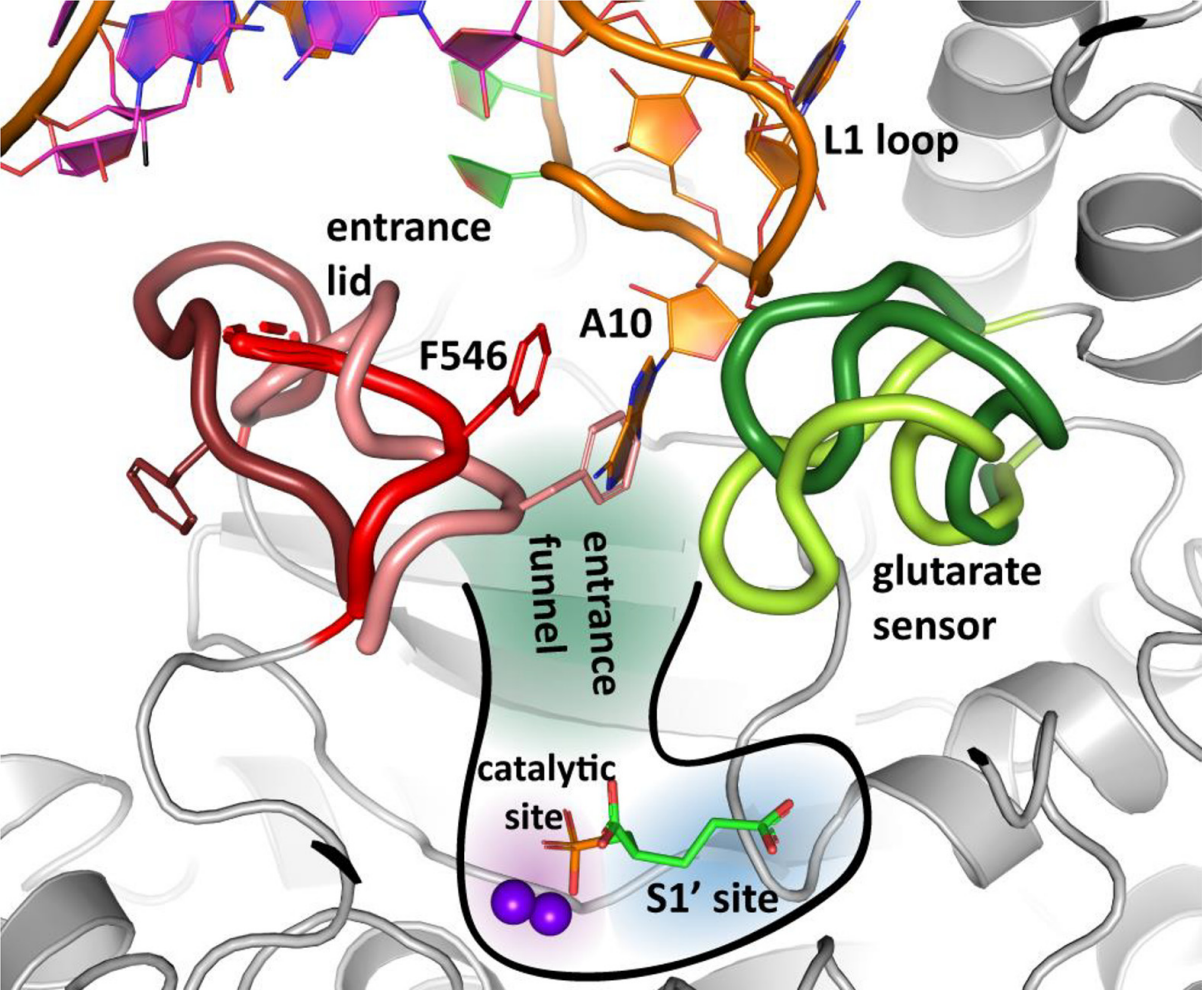
Flexibility of the entrance lid and the glutarate sensor and their conformational changes upon A9g binding. Superposition of PSMA complexes featuring different conformations of the entrance lid (amino acids 541–548, red shades) and the glutarate sensor (amino acids 692–704, green shades). PSMA is shown in gray cartoon with entrance lid and glutarate sensor highlighted as thick ribbons. The position and shape of the internal pocket, together with S1′-bond 2-PMPA (sticks), are sketched, the zinc ions are shown as purple spheres. The entrance lid can adopt a continuum of conformations between the ‘open’ (dark red; PDB code: 3BI1) and the ‘closed’ conformation (light red; PDB code: 3BI0). The glutarate sensor conformations are likely bimodal and oscillate between ‘withdrawn’ (S1′ site empty, dark green; PDB code 2C6P) and the ‘binding’ states (S1′ site occupied, light green; reported here). The A10 nucleobase bridges the entrance lid and glutarate sensor and locks them in the ‘semi-closed’ (red) and ‘binding’ (light green) conformations, respectively, effectively blocking the internal cavity of the enzyme.

The glutarate sensor (amino acids 692–704) and the entrance lid (amino acids 541–548) are two prominent flexible segments of PSMA that can adopt various conformations in response to the occupancy of the internal substrate-binding cavity of PSMA by different ligands, and both these segments are engaged by the A10 adenine ring of the L1 loop (Figures 3C and 4). In its extreme ‘closed’ and ‘open’ conformations, the entrance lid can either block or permit transport of solutes between the protein interior and the external milieu. Similarly, the glutarate sensor adopts a ‘binding’ or ‘withdrawn’ conformation when the S1′ substrate-binding pocket is either occupied or empty. By bridging the ‘binding’ and ‘semi-closed’ conformations of the glutarate sensor and entrance lid, respectively, the aptamer effectively locks PSMA in a defined state and blocks entering/release of a substrate/inhibitor into/from the internal pocket of the enzyme. It is interesting to note that neither ‘withdrawn’ (glutarate sensor) nor ‘closed’ (entrance lid) conformations are compatible with A9g binding to PSMA as these would result in steric clashes with the A10 of the L1 loop in the entrance funnel (Figure 4).

PSMA structural changes upon A9g binding are rather small and mostly confined to the residues directly interacting with the aptamer. The notable exception are two flexible segments—the entrance lid and the glutarate sensor—which adopt conformations compatible with the position of L1 loop in the entrance funnel, thereby enabling stacking of adenine A10 between residues F546 and A701 of the entrance lid and the glutarate sensor, respectively.

### The impact of S317 on PSMA binding to A9g

**Figure 5.**
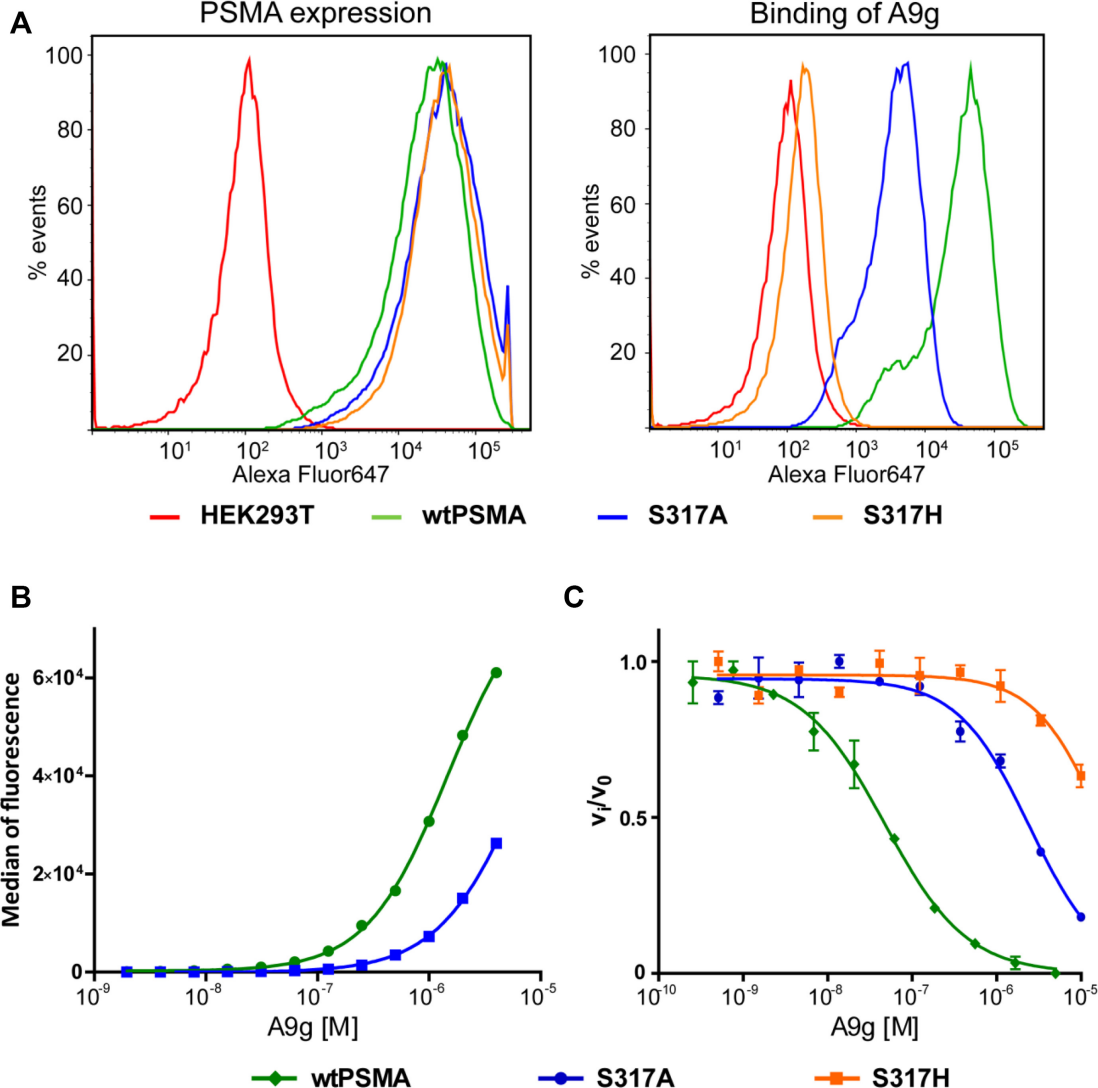
Effects of S317 mutations on PSMA/A9g interactions. (**A, left**) Flow cytometry analysis of expression levels of wtPSMA, and S317A and S317H mutants on the surface of transfected HEK293T/17 cells show comparable expression levels of all three PSMA variants. Cells were incubated with 40 nM 5D3 antibody and stained with Alexa Fluor647-conjugated goat anti-mouse secondary antibody. (**A, right**) Flow cytometry analysis of the A9g binding to HEK293T/17 cells expressing PSMA variants. Tested cell lines were labeled with 100 nM Alexa Fluor647-labeled A9g and analyzed by flow cytometry as described above. (**B**) Affinities of A9g toward wtPSMA and S317A cell lines. Dilution series of Alexa Fluor647-labeled A9g was incubated with transfected HEK293T/17 cells, analyzed by flow cytometry and median fluorescence plotted against A9g concentration. (**C**) Effect of S317 mutations on inhibitory potency of A9g. [^3^H]NAAG was used as a substrate and the cleaved product quantified by liquid scintillation. Three-fold dilution series of A9g were used to determine IC_50_ values against wild-type, S317A and S317H PSMA variants. The experiment was done in duplicate, and data are plotted as means ± standard error.

To test the importance of S317 for PSMA interactions with A9g and to confirm that the observed PSMA/A9g interaction interface is not a crystallographic artifact, we constructed two PSMA mutants, in which S317 was substituted with alanine and histidine, respectively. We hypothesized that while the S317A mutation would decrease the affinity of A9g for PSMA due to four missing hydrogen bonds, the S317H mutation would completely abolish A9g binding to PSMA due to the steric interference (Supplementary Figure S4A, C, D). To this end, we generated respective HEK293T/17 cell populations stably expressing either wild-type PSMA (wtPSMA) or its respective S317A and S317H mutants. PSMA expression levels at the plasma membrane of all three cell lines were virtually identical, as evaluated by flow cytometry using the PSMA-specific 5D3 monoclonal antibody (Figure 5A, left panel). At the same time, however, the fluorescence signal of the Alexa Fluor647-labeled A9g (AF647–A9g) was substantially weaker for the S317A variant and close to background for the S317H variant, thereby confirming our structural model (Figure 5A, right panel).

We also attempted to use flow cytometry to determine the binding affinity of the AF647–A9g toward individual PSMA mutants expressed at the HEK293T/17 surface. Unfortunately, as we were not able to reach the upper plateau of the binding curve, the binding constants could not be reliably derived from the flow cytometry data (Figure 5B). Consequently, we used an alternative approach, where the wild-type PSMA and the S317 mutants were expressed as soluble proteins in S2 cells, purified to homogeneity (Supplementary Figure S7) and A9g affinity for individual variants determined by an enzymatic competition assay with NAAG, the natural PSMA substrate (Figure 5C). In line with our structural predictions and the flow cytometry findings, the S317H substitution impaired PSMA-A9g interactions (IC_50_ around 20 μM), while the A9g inhibition constant for the S317A mutant was 2.4 μM, i.e. ∼50-fold higher than the wild-type PSMA (IC_50_ = 47 nM).

**Figure 6.**
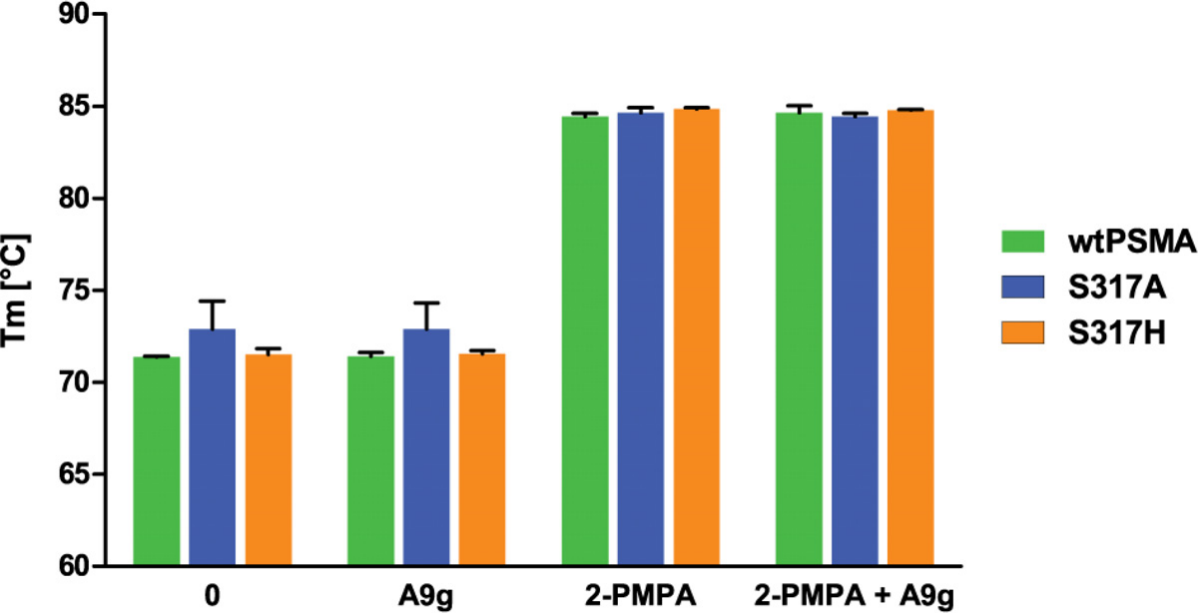
Thermostability of wtPSMA and S317 mutants using differential scanning fluorimetry (DSF). The denaturation curves of the purified extracellular part of wtPSMA and S317A and S317H mutants (1 μM) were measured for free enzymes, in the presence of 2-PMPA (10 μM), A9g (10 μM) or a mixture thereof. The melting temperatures (*T*_m_) were determined from the inflection points of melting curves. Experiments were done in duplicate, and data are plotted as means ± standard error.

As a complementary method to shed more light onto the mechanism by which A9g inhibits PSMA, we analyzed whether binding of A9g to PSMA stabilizes the active site and the overall fold and determined any differences between PSMA and S317A and S317H mutants. Here, we employed differential scanning fluorimetry (DSF) where we previously observed a substantial increase in the melting temperature (*T*_m_) of the PSMA upon binding of an inhibitor to its internal substrate-binding cavity. The increase in PSMA stability is likely due to ‘locking’ the enzyme in the ‘substrate-bound’ state, thus disabling rearrangement of the flexible parts of PSMA, especially the glutarate sensor required for substrate recognition (70). We confirmed this stabilizing effect on the PSMA as well as on both S317 mutants using 2-PMPA. However, incubation of PSMA with an excess of A9g did not show such an effect for any of the PSMA variants, suggesting that binding to the surface of PSMA is not sufficient for stabilizing the 3D fold and increasing the melting temperature of the enzyme (Figure 6).

Overall, S317 has been identified as a residue critical for high-affinity A9g binding and the structural data were corroborated by site-directed mutagenesis and ensuing *in vitro* and cell-based assays. Apparently, a replacement of S317 by histidine is sufficient to completely abolish PMSA–A9g interactions.

### Specificity of A9g for human PSMA

**Figure 7.**
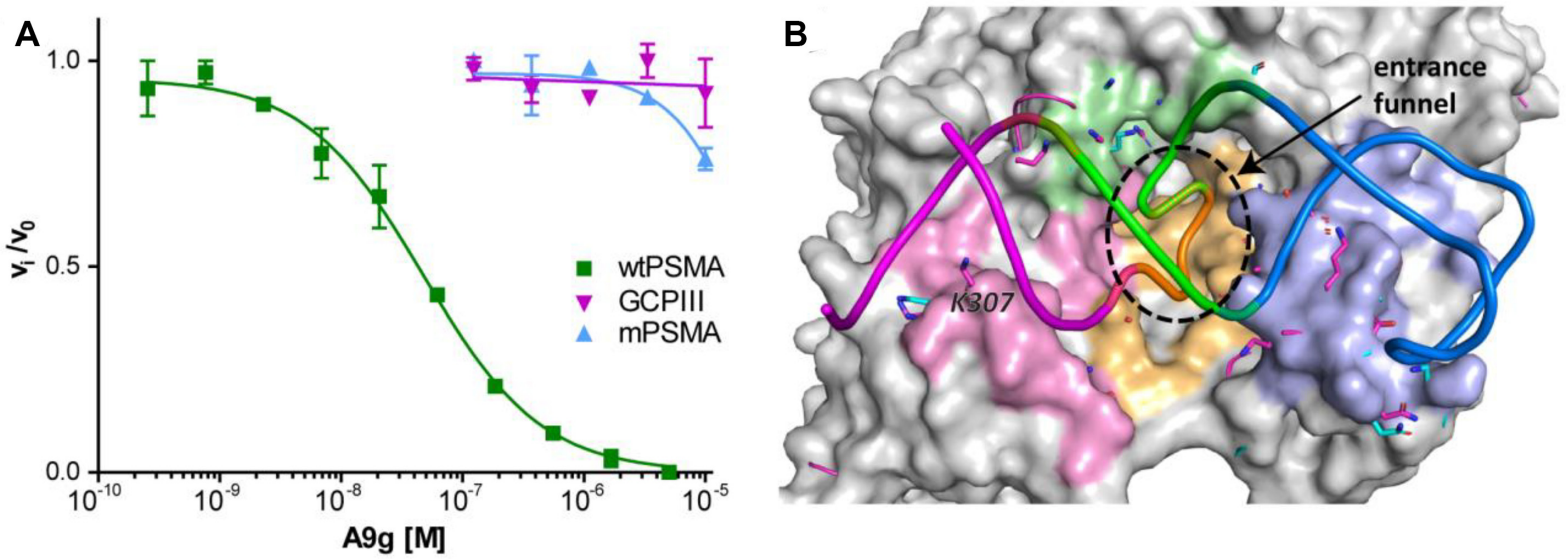
Comparison of A9g binding to human PSMA, GCPIII and mouse PSMA (mPSMA). (**A**) Enzymatic activity of purified human wtPSMA, mPSMA and GCPIII was determined in the presence of increasing concentrations of A9g using [^3^H]NAAG as a substrate. Experiments were done in duplicate and experimental points are plotted as means ± standard error. (**B**) Comparison of A9g-binding interfaces of PSMA, GCPIII and mPSMA. The three PSMA homologues were superposed on corresponding Cα atoms, human PSMA is shown in surface representation (gray) with all amino acids within 5 Å from A9g (color-coded ribbon: magenta S1 stem, orange L1 loop, green S2 stem, blue L2 loop) colored according to regions of A9g with which they interact. Corresponding amino acids from GCPIII and mPSMA are shown as magenta and blue sticks, respectively, and only side chains bigger than the matching PSMA residues are visible projecting from the PSMA surface.

Glutamate carboxypeptidase III (GCPIII), a close paralog of PSMA with high sequence similarity, is expressed in several human tissues (72). Many small-molecule ligands developed as PSMA-specific inhibitors bind to human GCPIII with nanomolar affinity (32), which could, in principle, hamper their use in clinical applications. To evaluate specificity and cross-reactivity of A9g against GCPIII and murine PSMA (mPSMA; a widely used experimental model), we have expressed and purified extracellular parts of GCPIII and mPSMA and indirectly quantified the affinity of A9g by determining inhibition of the NAAG-hydrolyzing activity of the enzymes in the presence of increasing concentrations of A9g. Our data revealed that A9g does not bind to GCPIII with any appreciable affinity and its binding to mPSMA is also very weak with the IC_50_ value over 100-fold higher than human PSMA (Figure 7A).

With the structure of the PSMA/A9g complex in hand, we compared A9g-interacting interfaces of PSMA, GCPIII (PDB code 3FF3) and mPSMA (structure generated using the SWISS-MODEL web interface (73) with the PSMA/2-PMPA complex as a template; PDB code 2PVW, (63)), to understand the exquisite selectivity of the aptamer for human PSMA. Sequence alignment and structural superposition of the three homologues are shown in Supplementary Figure S8 and Figure 7B, respectively. Of 47 residues at the PSMA/A9g interface, 31 and 39 are conserved for GCPIII and mPSMA homologues, respectively. Substitutions of two residues of GCPIII and mPSMA with smaller side chains or side chains missing critical functional groups (as compared to PSMA; Supplementary Figure S8, green) would likely disrupt existing PSMA/A9g interactions, thereby leading to lower A9g binding affinity for a given homologue. V548 and T612 of mPSMA, replacing F546 and K610 of PSMA, respectively, could serve as such examples. In the case of V548, the substitution would result in a loss of stacking between aromatic rings of F546 and the A10 nucleobase inserted into the entrance funnel of human PSMA, thereby disrupting one of the signature interactions. More importantly, side chains of several residues are bulkier than those of PSMA, thus forming a potential steric barrier blocking A9g binding to the target protein. Here, K307 of GCPIII, replacing S317 of PSMA, can serve as a prime example of such substitutions, further corroborating results of our mutagenesis studies.

These findings suggest that despite high sequence and structural homology between human PSMA, human GCPIII and mouse PSMA, A9g is highly specific for the former. This exquisite specificity contrasts with small-molecule inhibitors targeting the active site of the enzymes that are rather highly promiscuous and such cross-reactivity could, in principle, have adverse effects for the translation of PSMA-targeting small-molecule compounds into clinical use.

### Influence of PSMA-specific inhibitors on A9g binding

**Figure 8.**
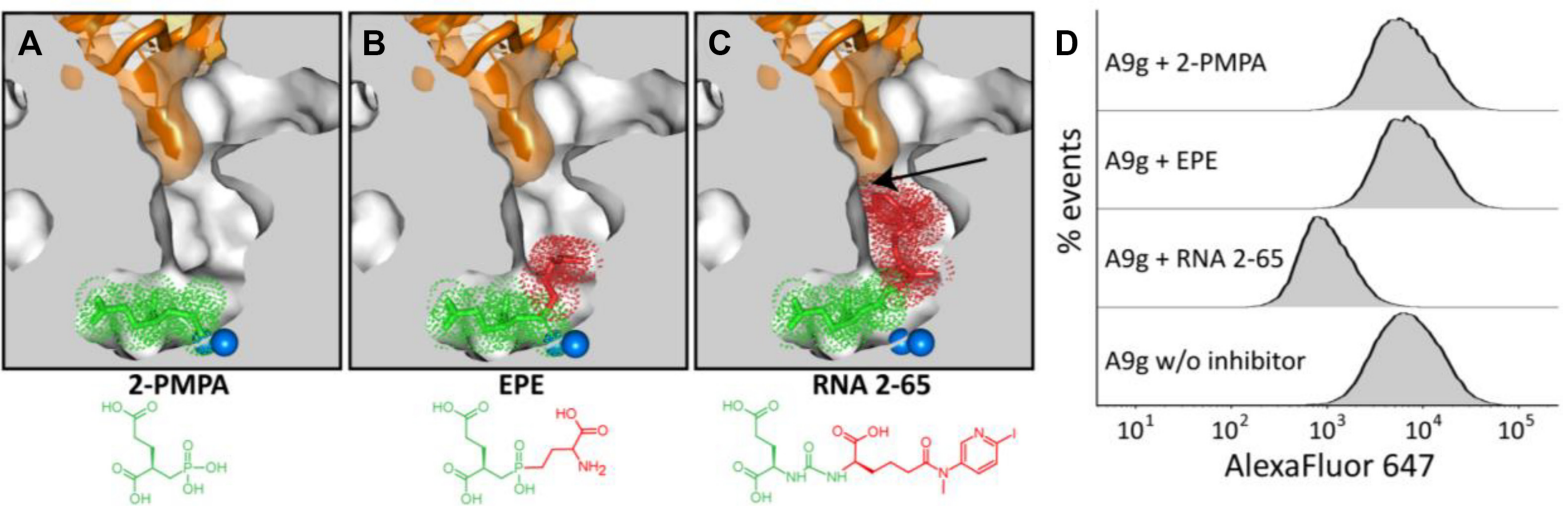
Inhibitors with bulky non-prime functionalities outcompete binding of A9g to PSMA. (**A–C**) Superposition of the PSMA/A9g complex and PSMA complexes with 2-PMPA (PDB code 2JBJ; panel A), EPE (PDB code 3BI0; panel B) and RNA 2–65 (PDB code 6H7Z; panel C). All complexes were superposed on corresponding Cα atoms. PSMA is shown in cross section (gray) revealing the internal inhibitor binding cavity (white). A9g is shown in semi-transparent surface representation (orange), the P1 part of inhibitors is colored red, and the zinc-binding group together with the P1′ part are colored green. A steric clash between A9g and the bulky nonprime functionality of RNA 2–65 is indicated by an arrow. The active-site zinc ions are shown as blue spheres. (**D**) Flow cytometry analysis of competition of A9g and PSMA-specific inhibitors. PSMA-positive PC3-PIP cells were preincubated in the presence of 1 μM inhibitor and 100 nM Alexa Fluor647-labeled A9g aptamer was added to the cell suspension. Aptamer binding was analyzed using an LSRFortessa flow cytometer.

The structure of the ternary PSMA/A9g/2-PMPA complex reported here suggests that A9g does not directly interfere with the binding of 2-PMPA in the S1 pocket of PSMA (Figure 8A). Furthermore, inspection of the PSMA/A9g complex suggests that other small-molecule PSMA ligands (including NAAG) can be accommodated by the enzyme even in the presence of bound A9g. On the other hand, larger PSMA ligands bearing bulky nonprime functionalities would compete with A9g binding predominantly by sterically blocking the insertion of A10 into the internal PSMA cavity. This hypothesis is illustrated by superposing structures of the PSMA-A9g complex with PSMA complexes harboring PSMA-specific inhibitors with either relatively small (EPE; Figure 8B) or bulky (RNA 2–65; Figure 8C) P1 moieties, respectively. To test this hypothesis, we carried out competition experiments using the PC3 cell line stably transfected with human PSMA (PC3-PIP cells). Cells were first incubated with a given inhibitor at a concentration of 1μM and binding of Alexa Fluor647–A9g was assessed by flow cytometry. In line with our structural predictions, we found that RNA 2–65 (a urea-based PSMA inhibitor with the bulky P1 moiety protruding from the active site into the entrance funnel) effectively blocked A9g binding to PC3-PIP cells, while EPE ((2*S*,3′*S*)-{[(3′-amino-3′-carboxy-propyl)-hydroxyphosphinoyl]methyl}-pentanedioic acid); a gem-diolate mimetic of NAAG) and 2-PMPA had no effect on A9g binding at the cell surface (Figure 8D).

## DISCUSSION

Only a handful of aptamer complexes can be found among the ∼3400 crystal structures of protein-nucleic acid complexes currently deposited in the PDB, despite the fact that aptamers have been developed for >1100 protein targets (74,75). The paucity of protein/aptamer structures reflects inherent difficulties in the crystallization of protein-nucleic acid complexes that are most likely linked to the inherent flexibility of the nucleic acid component(s). The results reported here thus represent one rare example of such structures.

Currently, small-molecule PSMA ligands, such as urea-based scaffolds, are used in clinic for prostate cancer imaging. These small-molecule compounds are popular for the ease of synthesis, high affinity toward PSMA and low production costs. At the same time, especially for therapeutic applications, two liabilities have been noted: (i) the accumulation of a tracer in salivary glands that could limit the therapeutic window and (ii) the potential to cross-react with GCPIII, a human paralog of PSMA with high structural similarity (31,32). In contrast to small molecules targeting the conserved active site of PSMA, macromolecular reagents, including antibodies, engineered binders (engineered protein scaffolds mimicking antibodies with a developed affinity for a desired target (76)) and aptamers are typically highly selective for a given target. Using recombinant human GCPIII and mouse PSMA, we tested whether A9g interacts with these members of the PSMA family using the NAAG hydrolysis activity assay. In line with the expected specificity of A9g, no inhibition was observed for variants other than human PSMA, thereby confirming its exquisite specificity (Figure 7).

In order to investigate systematically the common structural features governing protein–RNA aptamer interactions and binding-induced conformational changes upon the complex formation, we searched the Protein Data Bank (PDB; (77)) for the experimentally solved protein-RNA aptamer complexes (queries: ‘Contains protein: Yes’ and text ‘RNA aptamer’). Upon removal of complexes involving capsid proteins and short peptides, our search resulted in a total of 33 PDB entries covering 17 proteins that were then compared to corresponding experimental structures of apo proteins (Supplementary Table S3). Interestingly, our systematic analysis revealed that with two exceptions, protein conformational changes upon the protein–aptamer complex formation are minimal and are mostly localized to an interaction interface. For example, upon aptamer binding to the protein partner in the thrombin-RNA aptamer complex (PDB code: 3DD2; (78)) the global backbone RMSD of thrombin between the holo and the apo form is ∼0.2 Å, while residues at the binding interface can be displaced by 1–2 Å. Similarly, while the overall Cα RMSD between the apo and holo forms of hFc1 is only 1.1 Å in the human IgG-RNA aptamer complex (PDB code: 3AGV; (79)), the side chain of Lys340 is shifted by ∼4 Å to interact with the phosphate backbone of the RNA.

The above-mentioned exceptions, where the protein–aptamer complex formation results in pronounced global conformational changes of the protein partner, include the NF-κB (p50)_2_ homodimer–aptamer complex (PDB code 1OOA; (80)) and the HIV-1 Rev–RNA aptamer complex (PDB code: 6CF2; (81)). Compared to the NF-κB/DNA complex (PDB code 1NFK; (82)), upon aptamer binding NF-κB (1OOA) undergoes an allosteric conformational change resulting in an open conformation between its N- and C-terminal domains. This change is enabled by the presence of the flexible linker connecting the N- and C-terminal domains. Additionally, while the (p50)_2_ dimer wraps around a single central DNA molecule, each p50 monomer binds one molecule of the RNA aptamer. In the case of the HIV-1 Rev–RNA aptamer complex, the ARM helix and the proline-rich loop near the interaction interface undergo significant displacement in comparison with the apo structure of HIV-1 Rev (PDB code 2x7L (83); Supplementary Figure S9).

Overall, the existing structural evidence suggests that the global protein fold is typically preserved upon RNA aptamer binding and this finding is consistent with data on the PSMA/A9g complex reported here. The observed ‘structural rigidity’ likely results from the experimental procedures used for target-specific aptamer selection. Typically, an apo protein in its preferred, native conformation is used as a bait against an aptamer library and aptamer binders are enriched by successive rounds of selection and amplification under increasingly stringent conditions. It can be argued that this process will preferentially result in the selection of aptamer molecules structurally adjusted to the native target protein conformation rather than those substantially altering a native and thus energetically favorable target protein fold.

We next analyzed the structural features and binding characteristics of the 33 RNA aptamers listed in Supplementary Table S3 and the results of this analysis are shown in Supplementary Table S4. Structurally, 92% of aptamers contain helices, 78% feature stem–loops and 85% have at least a single flipped-out base. It is also interesting to note that only aptamers in 3AHU and 3HSB are single-stranded, whereas the remaining aptamers form duplexes. Thanks to its composition of a phosphate backbone, ribose rings and nucleobases, RNA aptamers are highly polar molecules. Upon binding to a target protein, they form a rich network of hydrogen bonds that play a critical role in RNA-protein interactions and define aptamer specificity. For the aptamer set analyzed here, there are on average 14 hydrogen bonds across the binding interface, with 7.8 and 8.1 hydrogen bonds involving nucleobases and backbones, respectively. In an alternative breakdown, 11.9 hydrogen bonds are related to nucleotides in loops versus 4.1 bonds related to nucleotides in helices. Clearly, while nucleobases and backbones contribute equally to hydrogen bonding with target proteins, interactions between protein residues and nucleotides forming loops are much more prevalent than nucleotides from stems and helices. In relation to this hydrogen-bonding pattern, the PSMA/A9g structure can thus be viewed as a typical representative of protein/RNA aptamer complexes with a predominant contribution of aptamer loops and comparable involvement of the phosphate backbone and nucleobases to protein binding. Apparently, a hydrogen bonding with nucleotides located in aptamer loops can be viewed as a hallmark of the protein-RNA aptamer interaction pattern.

Upon PSMA/A9g complex formation, nucleobases of the L1 loop flip out of the double helical central stem to interact with PSMA residues. It is interesting to note that analogous flipped-out conformations were found in 28 of the 33 (85%) aptamer-protein complexes listed in Supplementary Table S3. For example, there are three flipped-out aptamer bases in the thrombin-RNA aptamer complex (PDB code: 3DD2), but only one, C6, makes extensive contact with the protein, while the other two have minimal interaction with the enzyme. Similar conformations can be found in the repressor TetR–RNA aptamer complex (6SY4; (84)) and the human coagulation factor X-RNA aptamer complex 5VOE (85) and 3AGV. Unfortunately, as free aptamer structures corresponding to protein/aptamer complexes from Supplementary Table S3 are not available, it is not possible to draw any conclusions as to whether the flipped-out conformations are native to the aptamer in solution or enforced upon protein binding as suggested by our calculations from the PSMA/A9g complex. As aptamer structures comprising flipped-out nucleobases are much more prevalent, we assume that this distorted nucleobase conformation represents a typical binding motif in aptamer-protein interactions.

We have shown that A9g inhibits NAAG-hydrolyzing activity of PSMA with the IC_50_ value of 47 nM (Figure 5C). At the same time, competition experiments reveal that both NAAG and A9g can bind PSMA simultaneously. Taken together, these findings bring about an interesting question regarding the mechanism of the NAAG-hydrolyzing activity inhibition of PSMA by A9g. Our data suggest that while A9g does not directly outcompete NAAG binding to PSMA, it can directly block the entrance funnel leading to the active site of the enzyme, thereby precluding entry or release of the substrate or reaction products, respectively, effectively inhibiting the enzymatic activity of PSMA. Alternatively, the substrate hydrolysis can be abolished by limiting PSMA flexibility, most notably that of the glutarate sensor that is required for recognition of glutamate-like residues, by the A9g binding. In such a ‘frozen/locked’ state the enzyme might be unable to undergo structural changes associated with substrate binding and hydrolysis (e.g. glutarate sensor movement, changes in the distance between Zn^2+^ ions, arginine patch opening). The ‘lock-in’ of the enzyme in one conformation can be measured by the change in temperature stability of PSMA under various conditions. Indeed, the melting temperature of PSMA measured by differential scanning fluorimetry (DSF) increases by 10–13°C upon binding of 2-PMPA in the active site (Figure 6). On the other hand, no such effect has been observed for A9g (either for wtPSMA or the S317 mutants), thus indicating that no rearrangement of the active site occurred. This can be considered to be indirect evidence of the entrance funnel-blocking mechanism of PSMA inhibition by A9g.

In conclusion, data presented here provide a structural basis for the A9g engagement by human PSMA and reveal the lack of cross-reactivity of A9g towards human GCPIII, a close homologue of PSMA. Additionally, they reveal the mechanism of PSMA inhibition by A9g that stems from the blocking of the entrance site to the PSMA internal cavity. Our findings can be used for the future structure-assisted development of the next-generation PSMA-specific reagents.

## DATA AVAILABILITY

The final model coordinates and the corresponding structure factors for the PSMA/A9g complex have been deposited in the Protein Data Bank under accession code 6RTI.

## Supplementary Material

gkaa494_Supplemental_File
