# Supplementary material for: Structural basis of prostate-specific membrane antigen recognition by the A9g RNA aptamer
Source: Nucleic Acids Res. 2020 Jun 11;48(19):11130–45. doi: 10.1093/nar/gkaa494 (PMC7641732; doi:10.1093/nar/gkaa494)
Supplement: gkaa494_Supplemental_File [file gkaa494_supplemental_file.pdf]

Structural Basis of Prostate-specific Membrane Antigen Recognition by the A9g RNA Aptamer

Jakub Ptacek ,[1] Dong Zhang,[2] Liming Qiu,[3] Sven Kruspe,[4] Lucia Motlova,[1] Petr Kolenko,[1,5] Zora Novakova,[1], Shambhavi Shubham,[4] Barbora Havlinova,[1] Petra Baranova,[1] Shi-Jie Chen,[2,6] Xiaoqin Zou,[2,3,6] Paloma Giangrande,[4] and Cyril Barinka[1]

[1] Laboratory of Structural Biology, Institute of Biotechnology of the Czech Academy of Sciences, BIOCEV, Prumyslova 595, Vestec, 25250, Czech Republic

[2] Department of Physics and Astronomy, University of Missouri, Columbia, MO, USA

[3] Dalton Cardiovascular Research Center, University of Missouri, Columbia, MO, USA

[4] Department of Internal Medicine, University of Iowa, Iowa City, IA, 52242, USA

[5] Faculty of Nuclear Sciences and Physical Engineering, Czech Technical University in Prague, Brehova 7, Prague, 11519, Czech Republic

[6] Department of Biochemistry, Institute for Data Science and Informatics, University of Missouri, Columbia, MO, USA

Address correspondence to: Dr. Cyril Barinka, Institute of Biotechnology CAS, v.v.i., Laboratory of Structural Biology, Prumyslova 595, 25250 Vestec, Czech Republic; phone: +420-325-873-777; e-mail: cyril.barinka@ibt.cas.cz

Supplementary Table S1: Mutagenesis primer sequences. The point mutation site is flanked by complementary regions of 15 bps. Template sequence of PSMA (top row) and forward primer sequences are listed for both mutants; reverse primers are the reversed complement sequences of the forward primers. The mutated nucleotides are bolded.

wtPSMA	tcagcaccaccagatagcagctggagaggaag
S317A	tcagcaccaccagat**GC**cagctggagaggaag
S317H	tcagcaccaccagat**CA**cagctggagaggaag

Supplementary Table S2: Data processing statistics and structure refinement parameters. Values in parentheses refer to the highest-resolution shell.

**Data processing statistics**	
Space group	$P4_12_12$
Unit-cell parameters	
a, *b, c* (Å), α=β=γ=90°	121.1, 121,1, 216.3
Resolution range (Å)	105.66 – 2.20 (2.24 – 2.20)
No. of observations	1,039,676 (52,108)
No. of unique reflections	82,266 (4,051)
Data completeness (%)	100 (100)
Average redundancy	12.6 (12.9)
Average $I/\sigma(I)$	10.1 (2.0)
R_{merge}	0.164 (0.985)
$R_{p.i.m.}$	0.048 (0.284)
CC1/2	0.838 (0.578)
**Structure refinement parameters**	
No. of non-hydrogen atoms	
Glycoprotein	5872
Aptamer	974
Solvent	425
RMSD bond lengths from ideal (Å)	0.007
RMSD bond angles from ideal (degrees)	1.64
No. of Ramachandran outliers	1
R_{work}	0.192
R_{free}	0.217
R_{all}	0.195
PDB code	6RTI

Supplementary Table S3: Crystal structures of protein complexes with RNA aptamers deposited in the Protein Data Bank (1).

#	Protein Name	PDB Code (complexed with RNA aptamer)	PDB Code (free protein unless specified otherwise)
1	Glutaminyl tRNA Synthetase	1EXD	1NYL
2	NF-κB p50	1OOA	1NFK[1], 1SVC[1]
3	RNA Polymerase II	2B63	1NIK
4	Human IgG Chain C Region	3AGV	1FC1
5	E. coli Hfq Protein	3AHU, 3HSB	3QHS[2], 1HK9[2], 2YHT[2]
6	Thrombin	3DD2	2A2X, 3RLW
7	U1 Small Nuclear Ribonucleoprotein A	3EGZ, 3UCU, 3UCZ, 3UD3, 3UD4	1OIA
8	G Protein-Coupled Receptor Kinase 2	3UZS, 3UZT	1YM7, 4PNK
9	Ribosome-associated Protein L7AE-like	3V7E	2FC3[3]
10	BL3-6 Fab Antibody	4KZD, 4KZE, 4Q9Q, 4Q9R, 6B14, 6B3K, 6DB8, 6DB9	5EII[4]
11	Hen Egg White Lysozyme	4M4O, 4M6D	193L, 1LSG, 4YEO
12	Ribosomal Protein S8	4PDB	1SEI[5]
13	Chemokine CCL2	4R8I	4ZK9, 1DOK
14	Thrombin	5DO4	2A2X, 3RLW
15	Human Coagulation Factor X	5VOE, 5VOF	1IOE
16	HIV-1 Rev	6CF2	2X7L
17	Tetracycline Repressor Protein Class B	6SY4, 6SY6	4AC0

1. *Complex structures with bound DNA.*
2. *Homologous protein (45.3% sequence identity) without an RNA aptamer.*
3. *Homologous protein (41.5% sequence identity) without an RNA aptamer.*
4. *Homologous protein (92.8% sequence identity) without an RNA aptamer, with the mutations in the antibody located at the binding interface.*
5. *Homologous protein (86.9% sequence identity) without an RNA aptamer.*

Supplementary Table S4: Analysis of structural features and hydrogen-bonding pattern of known protein-RNA aptamer complexes.

PDB	Flipped-out bases in binding pockets?	Has helices?	Has stem-loops?	Total number of direct H-bonds	Number of H-bonds with nucleobases	Number of H-bonds with backbones	Number of H-bonds with nucleotides in loops	Number of H-bonds with nucleotides in helices
1EXD	Y	Y	N	40	16	24	29	11
1OOA	N	Y	Y	6	20	14	17	17
2B63	Y	Y	Y	11	1	10	4	7
3AGV	Y	Y	Y	4	8	4	10	2
3AHU	Y	N	Y	18	10	8	18	0
3HSB	Y	N	Y	25	16	9	25	0
3DD2	Y	Y	Y	16	5	11	12	4
3EGZ	Y	Y	N	21	17	4	17	4
3UCU	Y	Y	N	16	11	5	12	4
3UCZ	Y	Y	N	14	11	3	11	3
3UD3	Y	Y	N	11	9	2	9	2
3UD4	Y	Y	N	15	11	4	12	3
3UZT	Y	N	Y	3	1	2	3	0
3UZS	Y	Y	Y	3	1	2	2	1
3V7E	Y	Y	N	8	4	4	8	0
4KZD	Y	Y	Y	14	6	8	11	3
4KZE	Y	Y	Y	13	6	7	11	2
4Q9Q	Y	Y	Y	10	6	4	9	1
4Q9R	Y	Y	Y	12	6	6	11	1
6B14	Y	Y	Y	16	8	8	13	3
6B3K	Y	Y	Y	12	6	6	10	2
6DB8	Y	Y	N	13	7	6	10	3

6DB9	Y	Y	N	12	5	7	10	2
4M4O	N	Y	Y	7	4	3	7	0
4M6D	N	Y	Y	51	30	21	45	6
4PDB	N	Y	Y	14	1	13	3	11
4R8I	Y	Y	Y	13	7	6	12	1
5DO4	Y	Y	Y	16	5	11	13	3
5VOE	Y	Y	Y	11	9	2	11	0
5VOF	Y	Y	Y	12	9	3	12	0
6CF2	N	Y	Y	8	3	5	2	6
6SY4	Y	Y	Y	10	7	3	10	0
6SY6	Y	Y	Y	11	8	3	11	0

The H-bonds were identified by UCSF Chimera (http://www.cgl.ucsf.edu/chimera; (2)) with default parameters; only direct H-bonds between RNA aptamers and proteins are calculated. Clusters of the PDB structures sharing the same protein and RNA aptamer (but in different space groups) are grouped together and highlighted in different colors. For calculations, the multiple structures belonging to the same cluster are counted only once to avoid spurious results due to overcounting. In cases where structures within a cluster have varying numbers regarding a specific calculation, the average of the numbers would be used. For example, in the cluster containing 3UZT and 3UZS, the aptamer in only one has a helix thus, the contribution from this group to the calculation regarding the statistics of helices is 0.5.

Figure S1: The electron density map of the A9g aptamer.

Nucleotides are colored according to secondary structure elements: S1 stem – magenta, L1 loop – orange, S2 stem green and L2 loop cyan. The composite omit electron density map with simulated annealing (contoured at the 1.5 σ level) is shown as grey mesh. The map was calculated using PHENIX software (3,4). The two prominent regions interacting with PSMA are highlighted by red ellipses.

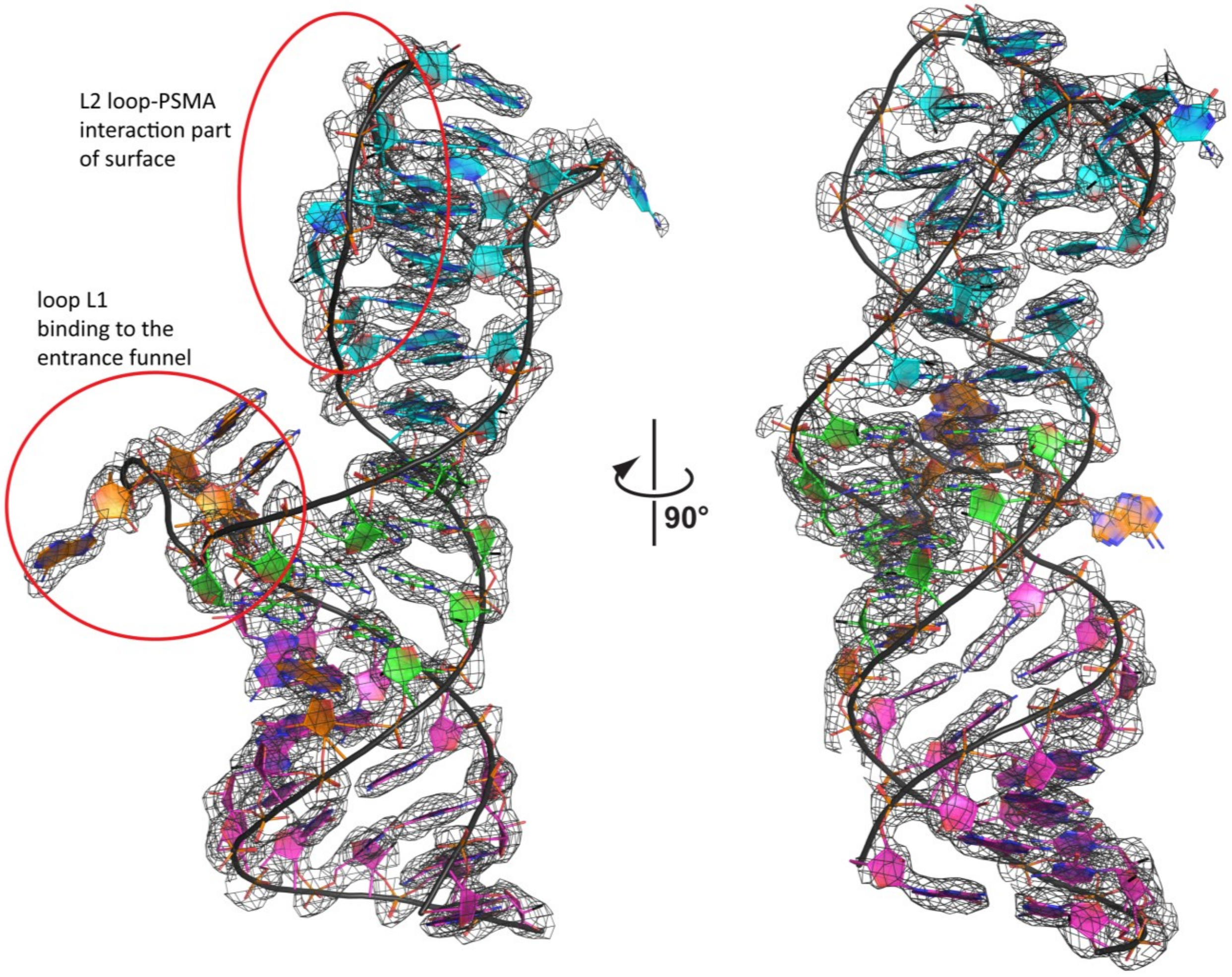

Figure S2: Details of noncanonical base pairing in the L2 loop.

The L2 loop comprises 16 bases (C16-A31), 13 of which are engaged in total eight non-canonical base-pairing interactions. Carbon atoms are colored green, orange, magenta, and cyan for C, A. U, and G, respectively. Nitrogen and oxygen atoms are colored blue and red, respectively, and the RNA backbone is shown as a black ribbon. Hydrogen bonds are shown as black dashed lines with interatomic distances in Å.

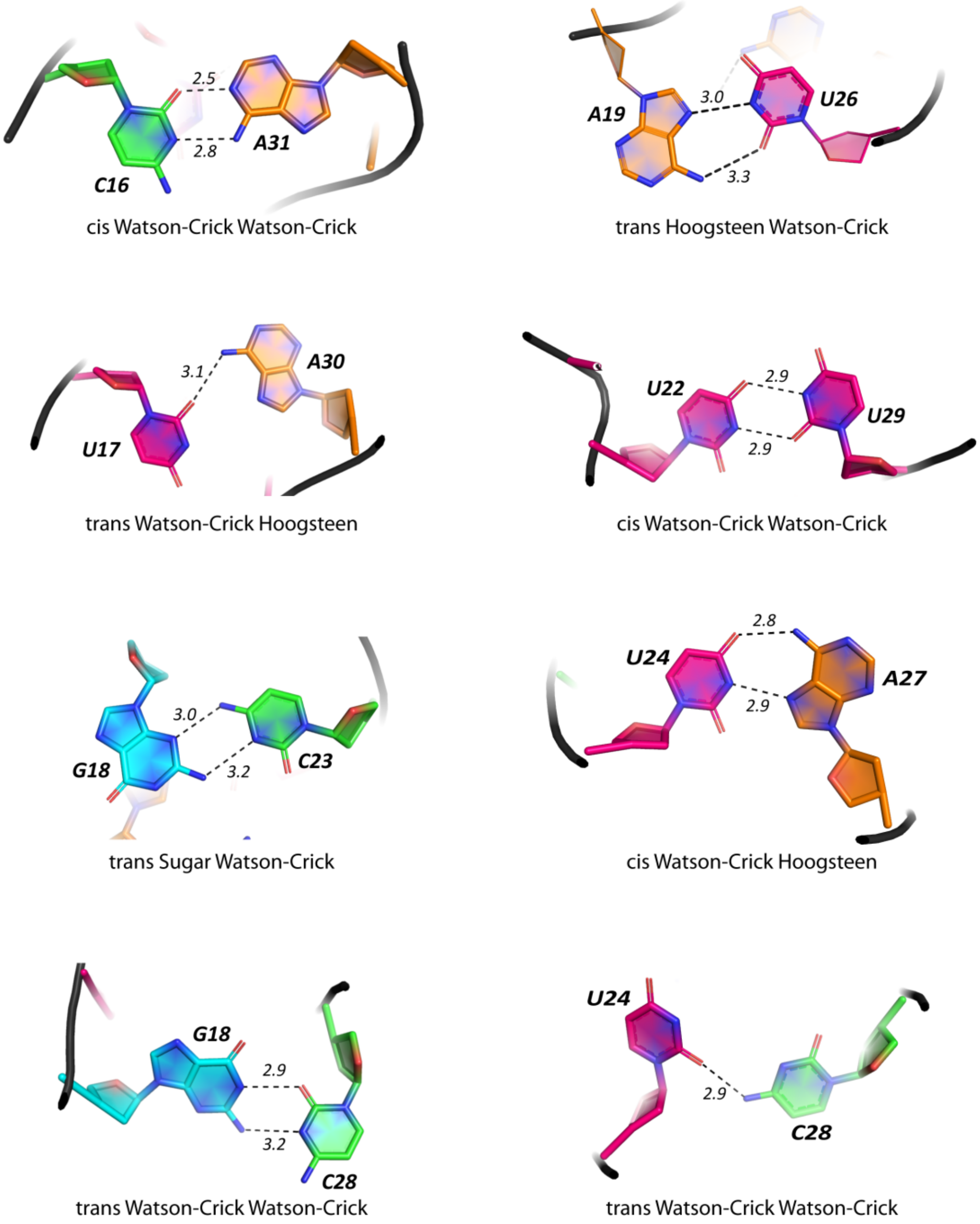

Figure S3: Crystal contacts of PSMA/A9g. Panel A: Stacking interactions between G1 and G1' of two A9g molecules are observed at crystallographic contacts between symmetry related molecules. **Panel B:** An alternative PSMA/A9g binding interface as generated by symmetry operators from the crystal structure. PSMA homodimer is shown in surface representations with individual monomers coloured blue and grey and the A9g is represented by the orange backbone illustration.

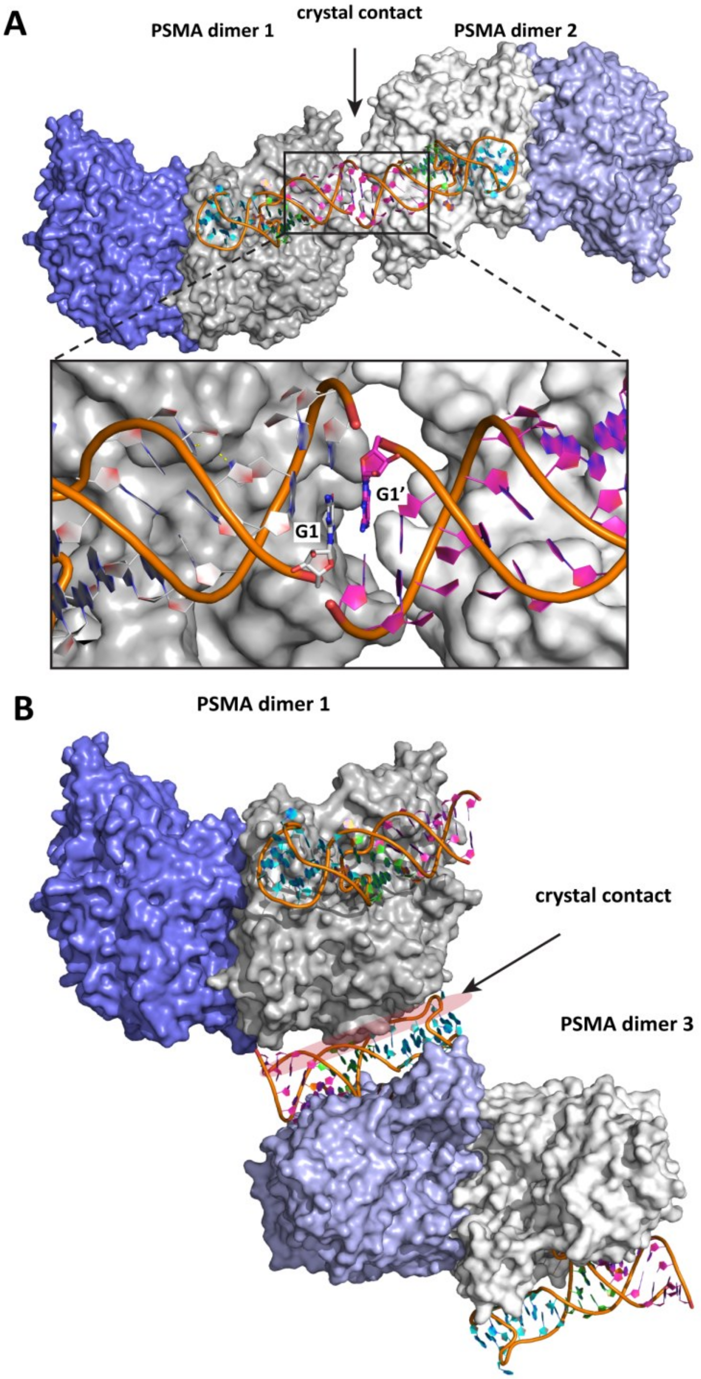

Figure S4: S317 plays a critical role in interactions between A9g and PSMA. Panel A: Ser317 plays a critical role in PSMA/A9g interactions by bridging C5 and U39 nucleotides. Putative hydrogen bonds are shown as black dashed lines (distances in Å). **Panel B:** Substitution of U at position 39 with G markedly decreases affinity of A9g to PSMA (5). The presented model shows that while this substitution can, in principle, increase the overall stability of free A9g by forming a Watson-Crick pair with C5, it would at the same time sterically clash with the side chain of S317 upon PSMA binding (arrow). **Panels C and D:** Models of S317A and S317H mutations. The four hydrogen bonds are missing in the S317A mutant, resulting in lower binding affinity of A9g for mutated PSMA. The presence of the bulky side chain of H317 is incompatible with the positioning of C5 and U39 bases (arrows), preventing thus the binding of A9g to PSMA. Serine and mutated residues are shown as sticks (carbon green, oxygen red, nitrogen blue), nucleotides are shown as lines with carbon atoms white, oxygen red, nitrogen blue and fluorine atoms black.

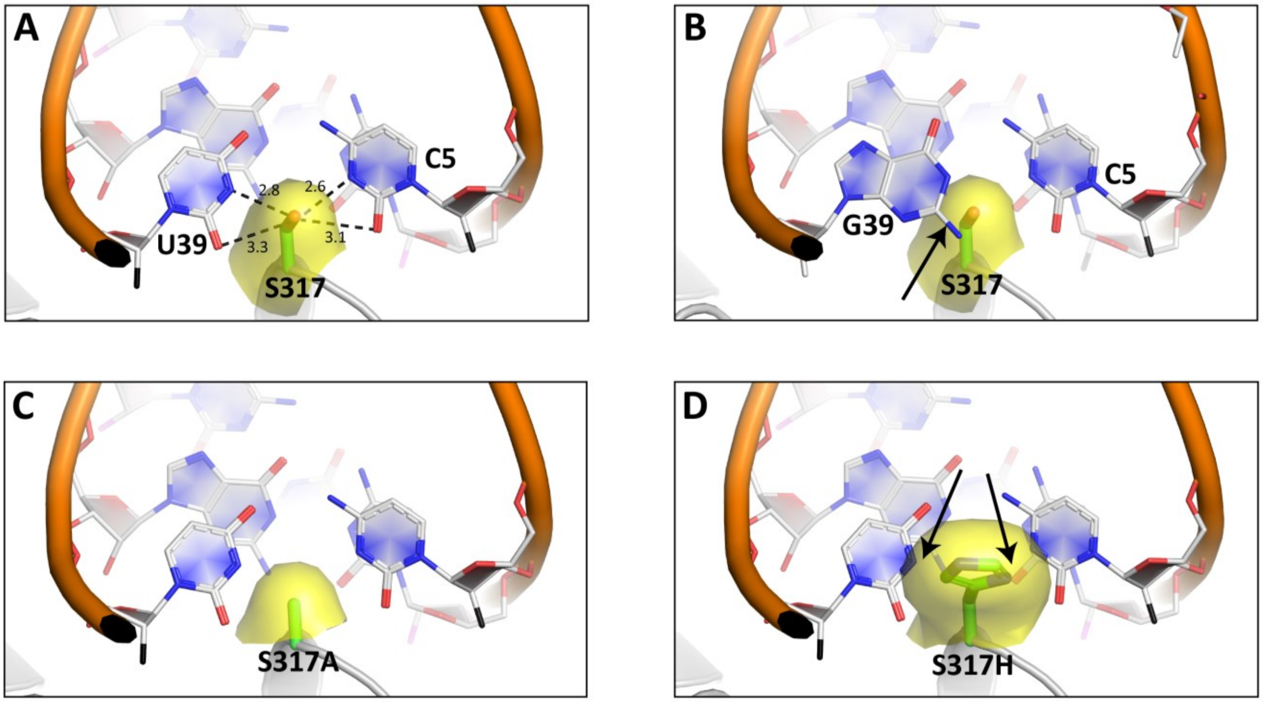

Figure S5: Positioning of the A9g L1 loop in the PSMA internal substrate binding cavity. The sharp turn of the loop allows flipping of adenine A10 out of the A9g double helix and enables its insertion to the entrance funnel of the substrate binding pocket of PSMA. The active site zincs are shown as blue spheres and green sticks represent 2-PMPA inhibitor bound in the active site.

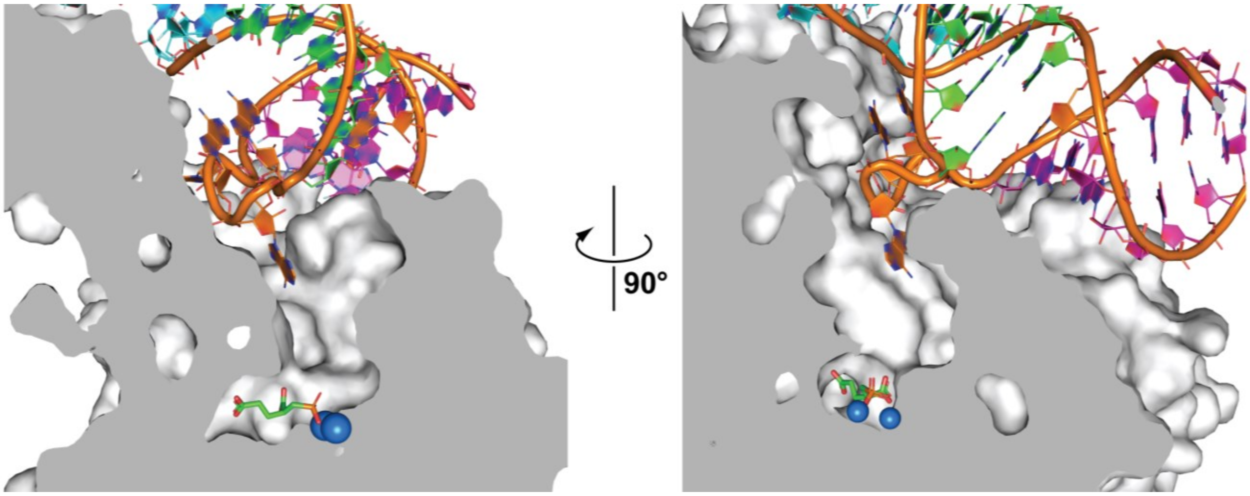

Figure S6: Non-polar interactions between adenines of the L1 loop and PSMA. Beside A10, which is stacked between F546 and A701 in the internal pocket of PSMA, two additional non-polar interactions with PSMA are observed for bases within the L1 loop. The nucleobase ring of A8 interacts with a surface patch formed by F186 (left) and a similar interaction is observed between A9 and I614 (right). The aptamer molecule is shown as an orange ribbon with bases as filled rings (carbons orange, nitrogens blue, oxygens red). PSMA is shown in a semi-transparent surface representation with carbon, nitrogen, and oxygen atoms colored white, blue, and red, respectively. Sidechains of F186 and I614 are shown as yellow sticks.

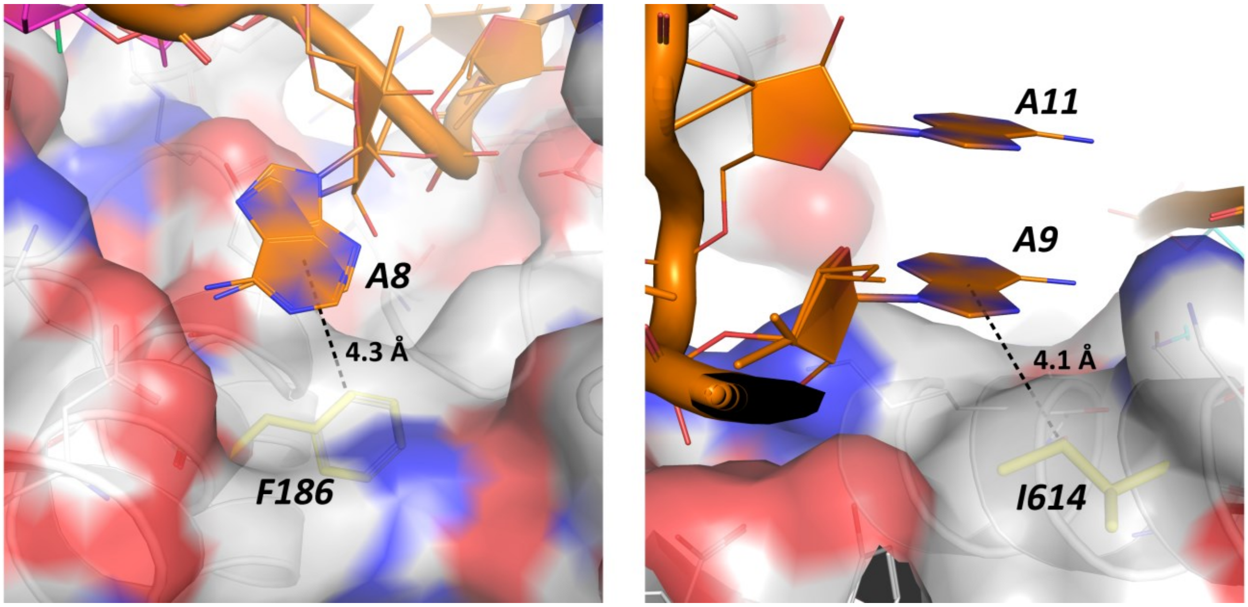

Figure S7: Purification of the extracellular part of wtPSMA and S317A and S317H mutants. Proteins were expressed with an N-terminal Strep-FLAG tag in Schneider's S2 cells and purified to homogeneity via StrepTactin affinity chromatography and size-exclusion chromatography. Purified proteins were analysed by SDS-PAGE 10% polyacrylamide gel.

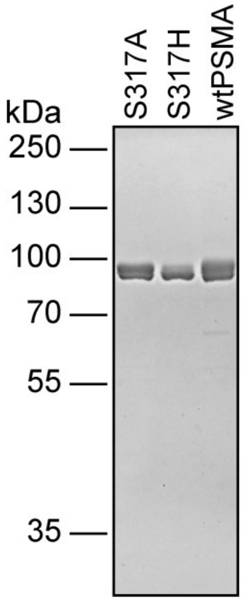

Figure S8: Sequence alignment of PSMA homologues. Sequences of extracellular domains of human PSMA, GCPIII and mouse PSMA were aligned using Clustal Omega (6). All amino acids forming PSMA-A9g interface are bordered. The positions are coloured in respect to human PSMA according to following criteria: conserved residues are marked cyan, substitutions with a potential to disrupt protein-RNA interactions are marked green and positions where amino acid in a homologue would introduce a steric clash are red.

```
human_PSMA                                                                    KSSNEATNITPK-H-N  57
human_GCPIII                                                                  KPLKETTTSVRYHQSI  47
mouse_PSMA                                                                    KPSNEATGNVSH-SGM  59

human_PSMA        MKAFLDELKAENIKKFLYNFTQIPHLAGTEQNFQLAKQIQSQWKEFGLDSVELAHYDVLL  117
human_GCPIII      RWKLVSEMKAENIKSFLRSFTKLPHLAGTEQNFLLAKKIQTQWKKFGLDSAKLVHYDVLL  107
mouse_PSMA        KKEFLHELKAENIKKFLYNFTRTPHLAGTQNNFELAKQIHDQWKEFGLDLVELSHYDVLL  119

human_PSMA        SYPNKTHPNYISIINEDGNEIFNTSLFEPPPPGYENVSDIVPPFSAFSPQGMPEGDLVYV  177
human_GCPIII      SYPNETNANYISIVDEHETEIFKTSYLEPPPDGYENVTNIVPPYNAFSAQGMPEGDLVYV  167
mouse_PSMA        SYPNKTHPNYISIINEDGNEIFKTSLSEQPPPGYENISDVVPPYSAFSPQGTPEGDLVYV  179

human_PSMA        NYARTEDFFKLERDMKINCSGKIVIARYGKVFRGNKVKNAQLAGAKGVILYSDPADYFAP  237
human_GCPIII      NYARTEDFFKLEREMGINCTGKIVIARYGKIFRGNKVKNAMLAGAIGIILYSDPADYFAP  227
mouse_PSMA        NYARTEDFFKLEREMKISCSGKIVIARYGKVFRGNMVKNAQLAGAKGMILYSDPADYFVP  239

human_PSMA        GVKSYPDGWNLPGGGVQRGNILNLNGAGDPLTPGYPANEYAYRRGIAEAVGLPSIPVHPI  297
human_GCPIII      EVQPYPKGWNLPGTAAQRGNVLNLNGAGDPLTPGYPAKEYTFRLDVEEGVGIPRIPVHPI  287
mouse_PSMA        AVKSYPDGWNLPGGGVQRGNVLNLNGAGDPLTPGYPANEHAYRHELTNAVGLPSIPVHPI  299

human_PSMA        GYYDAQKLLEKMGGSAPPDSSWRGSLKVPYNVGPGFTGNFSTQKVKMHIHSTNEVTRIYN  357
human_GCPIII      GYNDAEILLRYLGGIAPPDKSWKGALNVSYSIGPGFTGSDSFRKVRMHVYNINKITRIYN  347
mouse_PSMA        GYDDAQKLLEHMGGPAPPDSSWKGGLKVPYNVGPGFAGNFSTQKVKMHIHSYTKVTRIYN  359

human_PSMA        VIGTLRGAVEPDRYVILGGHRDSWVFGGIDPQSGAAVVHEIVRSFGTLKKEGWRPRRTIL  417
human_GCPIII      VVGTIRGSVEPDRYVILGGHRDSWVFGAIDPTSGVAVLQEIARSFGKLMSKGWRPRRTII  407
mouse_PSMA        VIGTLKGALEPDRYVILGGHRDAWVFGGIDPQSGAAVVHEIVRSFGTLKKKGRRPRRTIL  419

human_PSMA        FASWDAEEFGLLGSTEWAEENSRLLQERGVAYINADSSIEGNYTLRVDCTPLMYSLVHNL  477
human_GCPIII      FASWDAEEFGLLGSTEWAEENVKILQERSIAYINSDSSIEGNYTLRVDCTPLLYQLVYKL  467
mouse_PSMA        FASWDAEEFGLLGSTEWAEEHSRLLQERGVAYINADSSIEGNYTLRVDCTPLMYSLVYNL  479

human_PSMA        TKELKSPDEGFEGKSLYESWTKKSPSPEFSGMPRISKLGSGNDFEVFFQRLGIASGRARY  537
human_GCPIII      TKEIPSPDDGFESKSLYESWLEKDPSPENKNLPRINKLGSGSDFEAYFQRLGIASGRARY  527
mouse_PSMA        TKELQSPDEGFEGKSLYDSWKEKSPSPEFIGMPRISKLGSGNDFEVFFQRLGIASGRARY  539

human_PSMA        TKNWETNKFSGYPLYHSVYETYELVEKFYDPMFKYHLTVAQVRGGMVFELANSIVLPFDC  597
human_GCPIII      TKNKKTDKYSSYPVYHTIYETFELVEKFYDPTFKKQLSVAQLRGALVYELVDSKIIPFNI  587
mouse_PSMA        TKNWKTNKVSSYPLYHSVYETYELVVKFYDPTFKYHLTVAQVRGAMVFELANSIVLPFDC  599

human_PSMA        RDYAVVLRKYADKIYSISMKHPQEMKTYSVSFDSLFSAVKNFTEIASKFSERLQDFDKSN  657
human_GCPIII      QDYAEALKNYAASIYNLSKKHDQQLTDHGVSFDSLFSAVKNFSEAASDFHKRLIQVDLNN  647
mouse_PSMA        QSYAVALKKYADTIYNISMKHPQEMKAYMISFDSLFSAVNNFTDVASKFNQRLQELDKSN  659

human_PSMA        PIVLRMMNDQLMFLERAFIDPLGLPDRPFYRHVIYAPSSHNKYAGESFPGIYDALFDIES  717
human_GCPIII      PIAVRMMNDQLMLLERAFIDPLGLPGKLFYRHIIFAPSSHNKYAGESFPGIYDAIFDIEN  707
mouse_PSMA        PILLRIMNDQLMYLERAFIDPLGLPGRPFYRHIIYAPSSHNKYAGESFPGIYDALFDISS  719

human_PSMA        KVDPSKAWGEVKRQIYVAAFTVQAAAETLSEVA    750
human_GCPIII      KANSRLAWKEVKKHISIAAFTIQAAAGTLKEVL    740
mouse_PSMA        KVNASKAWNEVKRQISIATFTVQAAAETLREVA    752
```

Figure S9: Superposition of aptamer-bound and free HIV Rev protein. The free HIV Rev protein (PDB code 2X7L; cyan) was superposed on corresponding Cα atoms of the aptamer-bound ARM helix (PDB code 6CF2). Both ARM helix (red) and the proline–rich loop (brown) reveal pronounced conformational changes upon RNA aptamer binding. At the bottom of the figure a Fab fragment used for the Rev crystallization is shown.

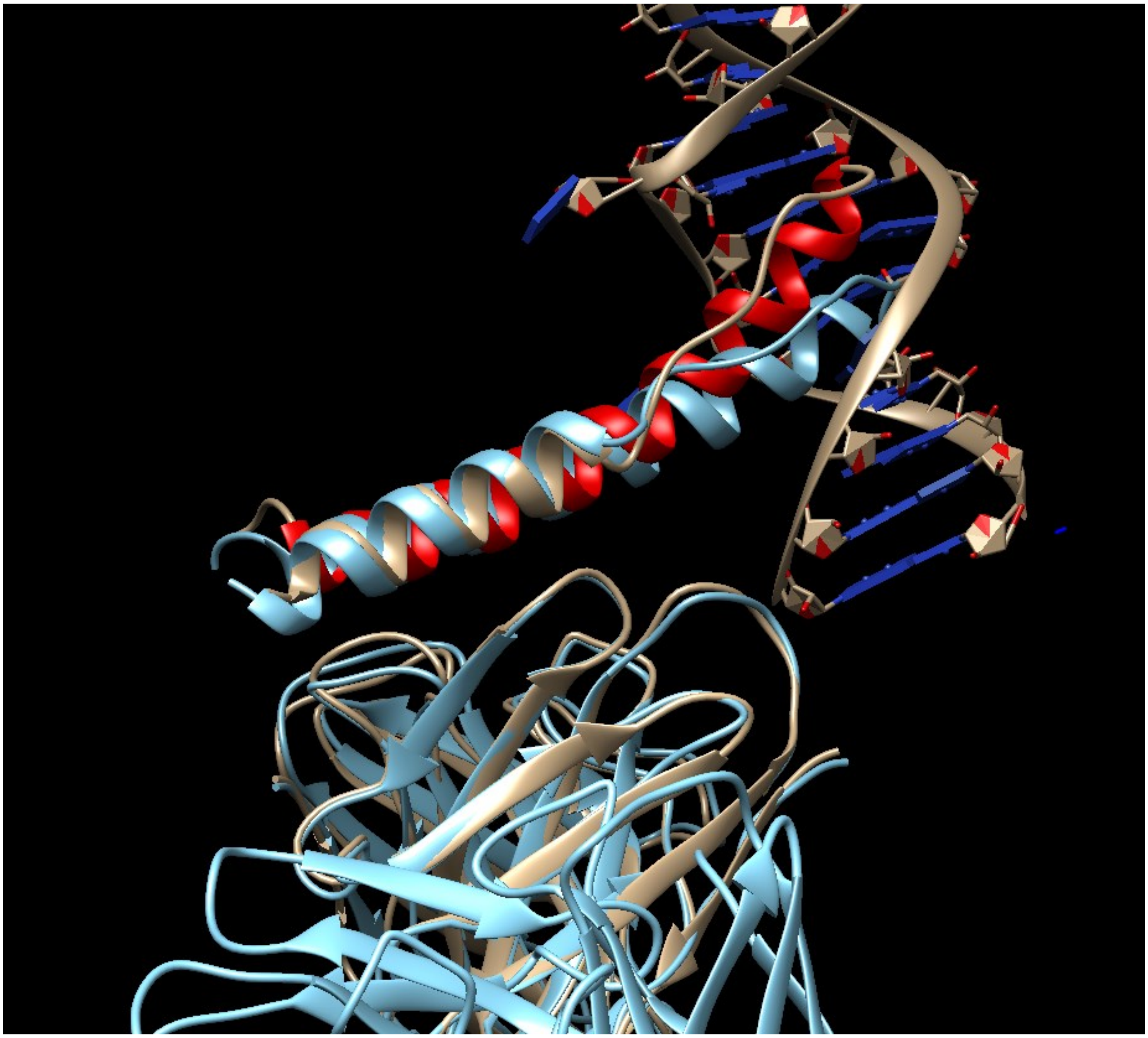

REFERENCES

1. Berman, H.M., Westbrook, J., Feng, Z., Gilliland, G., Bhat, T.N., Weissig, H., Shindyalov, I.N. and Bourne, P.E. (2000) The Protein Data Bank. *Nucleic acids research*, **28**, 235-242.
2. Pettersen, E.F., Goddard, T.D., Huang, C.C., Couch, G.S., Greenblatt, D.M., Meng, E.C. and Ferrin, T.E. (2004) UCSF Chimera--a visualization system for exploratory research and analysis. *Journal of computational chemistry*, **25**, 1605-1612.
3. Terwilliger, T.C., Grosse-Kunstleve, R.W., Afonine, P.V., Moriarty, N.W., Adams, P.D., Read, R.J., Zwart, P.H. and Hung, L.W. (2008) Iterative-build OMIT maps: map improvement by iterative model building and refinement without model bias. *Acta Crystallogr D*, **64**, 515-524.
4. Hodel, A., Kim, S.H. and Brunger, A.T. (1992) Model Bias in Macromolecular Crystal-Structures. *Acta Crystallogr A*, **48**, 851-858.
5. Rockey, W.M., Hernandez, F.J., Huang, S.Y., Cao, S., Howell, C.A., Thomas, G.S., Liu, X.Y., Lapteva, N., Spencer, D.M., McNamara, J.O. *et al.* (2011) Rational Truncation of an RNA Aptamer to Prostate-Specific Membrane Antigen Using Computational Structural Modeling. *Nucleic Acid Therapeutics*, **21**, 299-314.
6. Sievers, F., Wilm, A., Dineen, D., Gibson, T.J., Karplus, K., Li, W.Z., Lopez, R., McWilliam, H., Remmert, M., Soding, J. *et al.* (2011) Fast, scalable generation of high-quality protein multiple sequence alignments using Clustal Omega. *Mol Syst Biol*, **7**.
